# Transcriptomic Deconvolution of Neuroendocrine Neoplasms Predicts Clinically Relevant Characteristics

**DOI:** 10.3390/cancers15030936

**Published:** 2023-02-01

**Authors:** Raik Otto, Katharina M. Detjen, Pamela Riemer, Melanie Fattohi, Carsten Grötzinger, Guido Rindi, Bertram Wiedenmann, Christine Sers, Ulf Leser

**Affiliations:** 1 Knowledge Management in Bioinformatics, Institute for Computer Science, Humboldt-Universität zu Berlin, 10099 Berlin, Germany; 2 Department of Hepatology and Gastroenterology, Charité—Universitätsmedizin Berlin, Campus Virchow-Klinikum and Campus Charité Mitte, 13353 Berlin, Germany; 3 Laboratory of Molecular Tumor Pathology and Systems Biology, Institute of Pathology, Charité—Universitätsmedizin Berlin, 10117 Berlin, Germany; 4 German Cancer Consortium (DKTK), Partner Site Berlin and German Cancer Research Center (DKFZ), 69120 Heidelberg, Germany; 5 Section of Anatomic Pathology, Department of Life Sciences and Public Health, Università Cattolica del Sacro Cuore, 00168 Roma, Italy; 6 Anatomic Pathology Unit, Department of Woman and Child Health and Public Health, Fondazione Policlinico Universitario A. Gemelli IRCCS, 00168 Roma, Italy

**Keywords:** neuroendocrine neoplasm, neuroendocrine tumor, neuroendocrine carcinoma, NEN classification, deconvolution, machine learning

## Abstract

**Simple Summary:**

Rapidly growing neuroendocrine neoplasms (NEN) often defy easy classification by the pathologist. Machine learning approaches can improve the classification’s accuracy, but these generally require large amounts of training data. As tumor-based training data will remain sparse for very rare malignancies, such as NEN from the pancreas, we aimed for a machine learning-aided classification on the basis of the tumors’ similarity to non-transformed pancreatic cell types. We determined the relative contribution of the different healthy cell types to the transcriptome of each NEN and used the information to train a model for predicting the overall patient survival time, neoplastic grading, and carcinoma versus tumor subclassification. This approach does not use proliferation as a feature, since healthy pancreatic epithelial cell types do not proliferate. Hence, our approach is complementary to the established proliferation rate-based classification scheme, thereby providing additional criteria for a confident classification of ambiguous cases.

**Abstract:**

Pancreatic neuroendocrine neoplasms (panNENs) are a rare yet diverse type of neoplasia whose precise clinical–pathological classification is frequently challenging. Since incorrect classifications can affect treatment decisions, additional tools which support the diagnosis, such as machine learning (ML) techniques, are critically needed but generally unavailable due to the scarcity of suitable ML training data for rare panNENs. Here, we demonstrate that a multi-step ML framework predicts clinically relevant panNEN characteristics while being exclusively trained on widely available data of a healthy origin. The approach classifies panNENs by deconvolving their transcriptomes into cell type proportions based on shared gene expression profiles with healthy pancreatic cell types. The deconvolution results were found to provide a prognostic value with respect to the prediction of the overall patient survival time, neoplastic grading, and carcinoma versus tumor subclassification. The performance with which a proliferation rate agnostic deconvolution ML model could predict the clinical characteristics was found to be comparable to that of a comparative baseline model trained on the proliferation rate-informed *MKI67* levels. The approach is novel in that it complements established proliferation rate-oriented classification schemes whose results can be reproduced and further refined by differentiating between identically graded subgroups. By including non-endocrine cell types, the deconvolution approach furthermore provides an in silico quantification of panNEN dedifferentiation, optimizing it for challenging clinical classification tasks in more aggressive panNEN subtypes.

## 1. Introduction

The personalization of a patient’s treatment is the prime focus of the current translational research in biomedicine. It is defined as the adjustment of the treatment to patient-specific neoplastic characteristics and may identify more effective drug regimes, reduce side effects, and, ultimately, prolong a patient’s survival time while reducing costs [1,2]. Personalized treatment constitutes a particularly urgent need in rare cancer types with highly variable and unpredictable clinical courses, such as neuroendocrine neoplasms (NENs) and, more specifically, pancreatic neuroendocrine neoplasms (panNENs) [3]. Well-differentiated panNENs are referred to as neuroendocrine tumors (NETs) and typically exhibit a low (G1, G2) or, in rare cases, high (G3) proliferative index, as quantified by Ki-67 staining, with a median survival of the patients exceeding 10 years. NET patients are treated with a variety of approaches, only rarely including conventional chemotherapy [3]. In contrast, patients with poorly differentiated but highly proliferative neuroendocrine carcinomas (NECs) face a dismal prognosis of a few months and may profit from more aggressive, antiblastic therapies [4,5]. This diverse course of the disease stresses the need for a careful balancing of the treatments’ benefits and side effects and requires a precise characterization of each individual tumor.

The development of robust methods for characterizing patients with panNEN, or NENs arising elsewhere in the gastroenteropancreatic system (GI-NEN), is difficult for multiple reasons. First, panNEN and GI-NEN (GEP-NEN) are rare, which limits the availability of samples for research and model training purposes [5,6]. The age-adjusted incidence rate of well-differentiated GEP-NENs is estimated as 6.98 cases per 100,000 persons per year in the United States of America [6]. Second, the high degree of heterogeneity of GEP-NENs further reduces the availability of biomaterial for a specific subtype [3,7,8,9]. The frequency of subtypes is highly unbalanced: well-differentiated G1 (Ki-67 <3%) and G2 (Ki-67 3–20%) NETs occur distinctively more frequently than poorly differentiated NECs or G3 NET (Ki-67 >20%) [5,6]. Third, ambiguity with respect to the current morphologic NEC-versus-NET subtype classification can be very difficult to resolve. Even highly experienced pathologists encounter scenarios where a precise morphological classification of a GEP-NEN remains infeasible, prompting the inclusion of additional classification tools [10,11]. A precise classification, however, is mandatory for an effective personalization [1]. The existing in vivo classification methods, such as medical imaging, and tissue-based in vitro methods, such as Ki-67 immunohistochemistry staining (IHC), constitute the current gold-standard approaches [3,12]. Nonetheless, even these tools are limited in their ability to discern subtypes in samples with ambiguous morphologies and identical grades [11]. Furthermore, transcriptomic and epigenetic profiling clusters NETs predominantly based on their islet cell type resemblance or metastatic capacity rather than on their grading [13,14,15,16,17,18]. Therefore, novel tools for complementing and extending the current state-of-the-art in the case of ambiguity are urgently needed.

Over the last decade, machine-learning (ML) on next-generation sequencing (NGS) data has become the primary approach for the in silico characterization of neoplastic samples [19]. However, the training of robust and precise ML models, which can classify every subtype sufficiently well, requires the availability of suitable training datasets, encompassing large numbers of correctly classified samples with an unbiased and comprehensive coverage of the whole range of neoplastic diversity [20]. For panNENs and rare cancers in general, this poses a major challenge because the available training datasets are limited in both their sample sizes and subtype comprehensiveness. Such data scarcity can be countervailed with data augmentation in particular via the substitution of scarce data with more abundantly available training data, ideally without harming the predictive performance [21].

## 2. Materials and Methods

### 2.1. Overview of the Developed Framework

**Figure 1:**
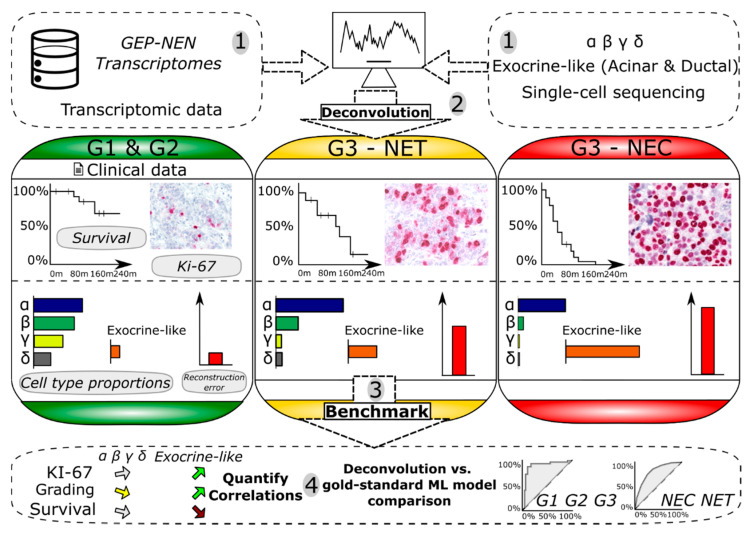
Overview of the framework which predicts clinically relevant panNEN and GEP-NEN characteristics based on a transcriptomic deconvolution. 1: Deconvolution algorithms are trained on different scRNA-based cell type datasets of healthy origin: endocrine-only and endocrine exocrine-like mixed. 2: The deconvolution *p*-values, cell type proportion predictions, and technical feature values are quantified for pancreatic and non-pancreatic NENs, different grading, and NEC and NET status. 3: Training of secondary ML models on the deconvolution results to predict clinically relevant properties of NENs from different benchmark datasets. 4: The deconvolution-trained ML model’s predictive power is compared to a baseline model. Additionally, the correlations between the cell type predictions with NEN grading, *MKI67*, and the survival time is calculated.

We have developed a two-step framework which first deconvolves panNENs based on their bulk-RNA-seq expression data, which are subsequently passed on to an ML algorithm which predicts the clinically relevant characteristics, see Figure 1.

### 2.2. Datasets

We procured three panNEN and 5 mixed, pancreatic, and non-pancreatic GEP-NEN datasets (see Table 1 and Supplementary Tables S1 and S2 for details on the type and source of the data and for their clinical properties). Deconvolution models were exclusively trained on scRNA data from pancreatic endocrine, exocrine, and adult human small intestinal stem cell types (HISC), respectively, which we refer to by the name of the first author, i.e., Baron [22], Lawlor [23], Segerstolpe [24], Tosti [25] or Haber [26] scRNA datasets. Epsilon cells were omitted due to their limited availability in the scRNA datasets. The exocrine-like cell type proportion consisted of the sum of the ductal cells and acinar cell type proportions for the Lawlor, Segerstolpe, and Baron datasets and of the sum of the acinar-i, acinar-s, acinar-reg+, ductal, and muc5b+-ductal cell types for the Tosti dataset.

Datasets were obtained and analyzed without any change to their data. The Fröhling, Riemer, and Scarpa datasets were procured as ‘.fastq’ files and their expression data were generated with best practice software pipelines. Where possible, *MKI67* expression data were obtained from the expression data by a look-up of the *MKI67* entry in the expression matrix. The Missiaglia dataset utilized a custom array which did not present with a *MKI67* annotated entry; however, Ki-67 staining levels were annotated for the samples which we used in lieu of the *MKI67* expression data.

The differential expressions results were corrected for multiple testing, utilizing the Benjamini–Hochberg method. The deconvolution results shown in Supplementary Table S3 were corrected for multiple testing with the Bonferroni method.

### 2.3. Bioinformatics Processing

Fastq read-based analyses of the Fröhling, Riemer, and Scarpa datasets were based on the human reference genome GRCh38 [31]. The reads were clipped and the adapters removed by the trim-galore software. Transcript per million (TPM) counts were utilized for analyses and generated by the Salmon software after an inspection of the raw data’s quality with fastqc [32]. Visualizations and findings other than differential expression were based on the TPM counts.

Differential expression analyses were conducted with the ‘DESeq2′ R-package and Love et al. best practice guidelines [33,34], whose design matrices were informed about the cohort and study membership of each sample to exclude potential batch effects during differential expression analysis. ‘Ggplot2′ and ‘ggbiplot’ were utilized for graphics generation. ‘Survival’, ‘sleuth’, ‘biomaRt’, and ‘RocR’ were further R-packages utilized for numeric analyses and the ‘stringR’ R-package for string-related operations [35,36,37,38]. The software ‘GSEA’, as provided by the Broad institute, Linux version 4.0.2 was utilized for the enrichment analyses. The survival curves were calculated with R-package ‘Survminer‘, version 0.4.8. The BSeq-sc [22] 1.0 R-implementation algorithm was acquired from cibersort.stanford.edu (accessed 23 November 2020). Beforehand, the most recent version 1.4 of the csSAM [39] (accessed 11 June 2020) R-package required to run Bseq-sc had been obtained from GitHub. The MuSiC algorithm version 0.1.1 was obtained from the GitHub repository github.com/xuranw/MuSiC (accessed 10 June 2021). The Moffitt et al. NMF algorithm [40] was trained (cell type signature matrix calculated) according to the specifications laid out in the corresponding publication which were replicated with the R-package ‘NMF’ version 0.2216.

### 2.4. Deconvolution Algorithms

We conducted preliminary studies and assessed multiple algorithms to the benchmark, including but not limited to SCDC [41] and UNDO [42], but ultimately narrowed down the selection to three algorithms. These three deconvolution algorithms were each trained on four pancreatic scRNA datasets: the CIBERSORT-based framework called BSeq-sc, procured from the Baron et al. publication [22], MuSiC [43], and Moffitt [40] (see Supplementary Table S4A and Figure 1). At any point in the manuscript where the term ‘BSeq-sc’ is utilized, we are referring to CIBERSORT [44] with its BSeq-sc framework [22]. Note that CIBERSORTx [45] is a related but different algorithm from BSeq-sc, a single-cell adaptation of CIBERSORT. Since CIBERSORT-based BSeq-sc and CIBERSORTx are related algorithms, we compared their performance with respect to the tasks relevant to this manuscript and have found their predictions to be comparable with a statistical significance at a great statistical power (see Supplementary Table S3C–E), which is why we do not list CIBERSORTx as an additional algorithm.

The ‘empirical *p*-value’ concept and its algorithmic implementation utilized to classify a deconvolution as statistically significant has been taken from the original Newman et al. publication and is explained in its methods section [44]. Neither normalization nor log-transformation was applied during the deconvolution and the number of permutations for the quantification of the empirical *p*-value of each deconvolution was set to 10^3^, where applicable. The cell type proportions were analyzed for the best performing combination of BSeq-sc [22] and Baron et al. scRNA training data [22]. We utilized the coefficients of the (ny) *v*-support vector regression underlying BSeq-sc for the cell type proportion predictions. Relative cell type proportion predictions were generated by dividing all the absolute cell type proportion coefficients by the overall sum of all the coefficients.

Before the models were trained, a differential expression analysis was performed to identify 800 cell type-specific marker genes whose expression was significantly higher in a given cell type compared to all other cell types, utilizing the limma R package [46]. Note that models consisting of multiple cell types were thus trained on an aggregate of about 4000–5000 genes since each cell type created a partly unique set of marker genes. The number of 800 genes per cell type was selected as a trade-off between the performance and computational resource requirements. We as well lowered the number of genes in multiples of 2 down to 50 marker genes per cell type while benchmarking the performance to verify that a lower number of genes would not result in a better performance, which could result from a reduced multicollinearity.

Pathologists classified the Riemer and Fröhling datasets’ tissue sections overall as suitable for RNA sequencing. We determined the extent of immune infiltration or other stromal tissue components via the application of the ESTIMATE algorithm to the datasets that required processing (Fröhling, Riemer, and Scarpa) and found the tumor purity comparable between the datasets (range cohort means tumor purity ~80% for Fröhling and up to 95% for Riemer) [47].

We chose BSeq-sc [22], MuSiC [43], and Moffitt et al. [40] due to their proven ability to deconvolve either healthy pancreatic tissue (BSeq-sc, MuSiC) or cancerous exocrine pancreatic tissue (Moffitt et al. [40]). Subsequently, we identified the combination of training scRNA dataset and deconvolution algorithm, whose predictions were most suited by comparing the stability, significance, and statistical power of the resulting correlations. The Pearson product–moment correlations of the relative fractions and the *MKI67* levels were subsequently calculated to compare the performance to predict the sample grading and overall patient survival time.

We ascertained that the marker genes used for the deconvolution models were approximately equally expressed in the non-pancreatic and the pancreatic NENs tissues by conducting a differential expression analysis, followed by the determination of the intersect of the significantly differentially expressed genes between the marker gene signatures. We found that only 4% of the exocrine-like signature genes showed a differential expression activity between pancreatic and non-pancreatic tissue and therefore deemed the exocrine-like cell type proportion to be free of any tissue-related bias.

We furthermore ensured that the exocrine-like marker genes were not associated with proliferation activity by calculating their overlap with the proliferation-specific GO-annotation gene set CELL PROLIFERATION GO 0008283. We found that the overlap amounted to only 5% and therefore did not constitute a confounding factor for the deconvolution, i.e., we deem the deconvolution results to be proliferation rate-independent. The ML models which predicted the clinical characteristic were exclusively trained on deconvolution-derived results, such as the relative cell type proportions, which did not contain proliferation rate-informed features.

### 2.5. Machine Learning and Survival Time Prediction Test

We first generated a ‘baseline’ model containing gene expression data, which included the expression levels of *MKI67*. *MKI67* is crucial because the staining levels of its protein Ki-67 are—among other factors—a key measurement for pathologists when classifying panNEN. The current clinical practice assigns Grade 1 to NEN with Ki-67 positive tumor cell fractions <3%, Grade 2 to NEN with Ki-67 fractions from 3 to 20%, and Grade 3 to NEN with Ki-67 fractions >20%. The baseline model therefore serves as a proxy to classify panNENs with special emphasis on *MKI67*, while the additionally generated deconvolution model serves to assess the performance of a model without knowledge of *MKI67* and instead contains deconvolution results assumed to be informative with respect to a panNENs clinical properties.

The baseline model was trained on the expression data of 3474 genes and not altered or batch corrected. These 3474 genes were chosen on the grounds that they were shared by all six panNEN datasets, which allowed for the generation of a model that was representative for all the datasets. All panNEN datasets were merged into a matrix and random samples (columns) of the matrix were selected for the model generation process without balancing for study membership during the hold-out and the training data selection process. The removal of multi-collinearly correlated genes was conducted as well as correction for class-imbalance during the training time by selecting classes such that they were balanced.

We applied a softmax multi-class logistic regression algorithm trained by the PyCcaret package for both the binary and ternary grading standards [48]. As features of the deconvolution model, we used the root-mean-squared error (RMSE) of the transcriptomic reconstruction, the correlation r value, reconstruction *p*-value, and the cell type proportions depending on the model (endocrine-only, or endocrine and exocrine-like combined/mixed) as features. The model’s architecture followed the automatic model and feature-tuning approach of the utilized PyCarret software (version 3.0). A more detailed description of the deconvolution model’s output features is provided in Supplementary Text S2: Supplementary Methods.

For each task, we trained on 80% of the data and predicted on the 20% hold-out data that were not observed by the ML model during the training time. The correlation was between the z-transformed marker gene signature (a centroid) and the deconvolved transcriptome.

The Califano et al. [18] dataset did not provide grading information. Regardless, we could use this dataset as an unsupervised deconvolution cohort and found that the distribution of the resulting deconvolution models *p*-values were comparable to those of all other panNEN and GEP-NEN datasets.

The thresholds for the Cox hazard-ratio tests subgroups were determined by averaging the aggregated gradings’ cell type proportions or *MKI67* levels, e.g., aggregated values were summed up and divided by two to obtain the distinguishing threshold between the ‘low’ and ‘medium’ subgroups and an analogous approach was taken for the ternary design. The grading survival statistics were utilized ‘as-is’ without any alteration.

The Riemer dataset encompassed morphologically ambiguous samples with a conflicting classification between the study pathologists. These were defined as ‘NEC-like’ or ‘NET-like’ based on their similarity to histomorphological unambiguous NET or NEC samples in supervised clustering using the pNETassigner signature, established by Sadanandam et al. [15] as a transcriptome-based classification scheme. To ascertain that our model’s ability to predict NET or NEC features was not dependent on this allocation of ambiguous samples by non-standard criteria, we duplicated the analyses while excluding all the morphologically ambiguous samples. We did not observe a significant change in any NEC/NET-related prediction performances. Hence, we retained ambiguous samples to increase the sample size in particular for the rare panNECs.

### 2.6. Data Availability

All data with the exception of the Riemer et al. dataset (Riemer, P.; Otto, R.; Detjen, K.M., et al. Correspondence to pamela.riemer@charite.de, Laboratory of Molecular Tumor Pathology and Systems Biology, Institute of Pathology, Charité Universitätsmedizin Berlin, 10117 Berlin, Germany. Manuscript in preparation) are publicly available. The Riemer dataset can be accessed under the ID EGAD00001006657 on the EGA online repository.

### 2.7. Code Availability

The R-package ‘artdeco’, which contains the framework, is freely available on GitHub: https://github.com/RaikOtto/artdeco (accessed on 1 November 2022). The CIBERSORT (required for BSeq-sc) and MuSiC algorithms have to be installed separately due to the license restriction of these third-party algorithms. A source code different from the framework is available upon request.

## 3. Results

### 3.1. Creation of a Deconvolution Machine Learning Model in the Absence of Neoplastic Training Data

Our approach to the classification of pancreatic neuroendocrine neoplasms (panNENs) in the absence of suitable training data is centered on the hypothesis that a transcriptomic deconvolution is informative with respect to the clinical characteristics. We corroborated the hypothesis by benchmarking the approach on both panNENs and the wider group of gastroenteropancreatic NENs (GEP-NENs). We structured the machine learning (ML) software as a two-step framework whose first step incorporated deconvolution ML algorithms, while the second step consisted of panNEN ML classification algorithms. The first step deconvolves panNENs into relative cell type proportions, a step which only requires the training of the deconvolution models on the data of healthy tissue. The second step subsequently predicted the clinical characteristics of panNENs based on the deconvolution results (see Figure 1). Importantly, the framework did not require the scarcely available training data of neoplastic panNEN tissue and thus explicitly addresses the ubiquitous lack of sufficient training data in rare cancers.

The implemented analysis process commences by instructing a deconvolution ML model to differentiate between types of healthy, single-cell RNA (scRNA)-sequenced cells based on the expression of the respective marker genes which distinguish the cell types from each other. Next, the framework deconvolves panNEN transcriptomes to quantify the relative cell type proportions that a panNEN consists of. Here, transcriptomic deconvolution is defined as a non-negative matrix factorization which aims to reconstruct a given matrix with a signature and a proportion vector, i.e., an estimate which cell types make up the sequenced convolute. Along with the cell type proportions, technical features such as a panNEN sample-specific reconstruction error and an empirical deconvolution *p*-value with respect to the quality of the reconstruction are obtained. Third, the deconvolution output is utilized as the input data for a second ML model, which is trained to characterize panNENs and non-pancreatic GEP-NENs with respect to their grading, carcinoma (NEC) versus well-differentiated tumor (NET) status, and the overall patient survival time. For the evaluation, we compared the predictive capacity of this proliferation rate-agnostic ML model to the performance of a baseline ML model trained on the mRNA expression and *MKI67* proliferation rate biomarker data from neoplastic tissue.

### 3.2. Deconvolution Algorithms, Cell Type Models, and Evaluation Datasets

The effectiveness of a deconvolution-based approach critically depends on the choice of the deconvolution algorithm and the underlying scRNA cell type training data [41]. We systematically evaluated three state-of-the-art deconvolution algorithms: BSeq-sc (based on CIBERSORT) [22,44], MuSiC [43], and non-negative matrix factorization (NMF) as applied by Moffitt et al. [40] on pancreatic adenocarcinoma (PDACs). We furthermore identified three scRNA studies with a focus on the single-cell sequencing of islet cell preparations, which contained endocrine and admixed exocrine cell types, and one single nuclei RNA study aimed at the unbiased representation of the full repertoire of pancreatic cell types. We refer to these datasets by the names of their first authors: Baron [22], Lawlor [23], Segerstolpe [24], and Tosti [25] (see Supplementary Table S1).

We considered two different cell type models for the deconvolution of the data from neoplastic tissue into cell type proportions. The endocrine-only cell type model consisted exclusively of the endocrine cell types of alpha (α) to delta (δ). The second “mixed” model, in turn, contained all the cell types of the endocrine-only model and, additionally, the exocrine acinar and ductal cell types. Technically, the latter two were aggregated into a single artificial cell type called ‘exocrine-like’ by summation over the acinar and ductal proportions. The reasons for designing a mixed endocrine/exocrine model were: (i) the trans-differentiation of endocrine to exocrine cell types and vice versa occurs in mouse models of pancreatic injury, regeneration, and carcinogenesis [49,50,51], (ii) panNEC share mutational profiles with pancreatic adenocarcinoma [52,53,54] and may exhibit areas of pancreatic adenocarcinoma [12], (iii) the DNA methylation analyses in panNEC suggested acinar cells as the cell of origin [55], and (iv) adult pancreatic stem or progenitor-like cells are proposed to reside in the exocrine compartment [56,57,58,59]. Three panNEN and five GEP-NEN datasets were deconvolved with twelve combinations of the deconvolution algorithm and scRNA training dataset to uncover whether a transcriptomic deconvolution of panNENs and non-pancreatic GEP-NENs was possible and to identify which combination was most effective. To that end, we obtained 356 panNEN and 157 GI-NEN samples for a total of 513 GEP-NENs to benchmark their deconvolution (see Supplementary Table S2). Of these, 22 were organoid cultures and the remaining 491 samples were patient tissues. In the following, we refer to the datasets by the name of their publication’s corresponding authors, namely Califano [18], Diedisheim [17], Fröhling [28], Missiaglia [29], Riemer (Riemer, P.; Otto, R.; Detjen, K.M., et al. Correspondence to pamela.riemer@charite.de, Laboratory of Molecular Tumor Pathology and Systems Biology, Institute of Pathology, Charité Universitätsmedizin Berlin, 10117 Berlin, Germany. Manuscript in preparation), Sadanandam [15], Sato [30], and Scarpa [16]. All panNEN datasets provided information with respect to the neoplastic grading and NEC or NET status with the exception of the Califano dataset, which only provided annotation information with respect to the primary or metastasis status of a panNEN. The Riemer and Scarpa datasets annotated the disease-related survival times for the sequenced samples. Additionally, 89 non-neoplastic samples were grouped into the Fadista [27] dataset and were deconvolved to obtain a deconvolution *p*-value baseline.

**Figure 2:**
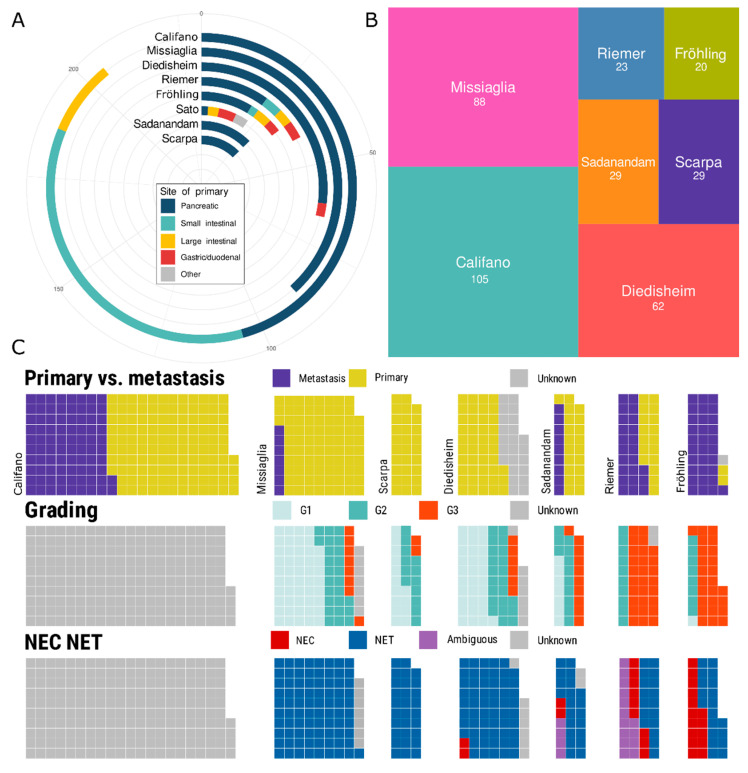
Overview of the benchmarked pancreatic and non-pancreatic NEN datasets. (**A**): Stratification with respect to the site of the primaries of the benchmarked 513 pancreatic and non-pancreatic NEN from eight studies. (**B**): Relative and absolute contributions of the seven panNEN datasets with respect to the set of 356 benchmarked panNENs. (**C**): Available metadata annotation for the seven panNEN datasets. Primary versus metastasis annotation is widely available while grading and NEC versus NET status are less frequently available while pancreatic NECs are provided by four datasets. Patient overall survival time data were available for the Riemer and Scarpa datasets.

Grading annotation was available for 238 panNENs and NEC or NET status for 227 panNENs. The subgroup of G3 panNENs comprised 30 NETs and 16 NECs (see Figure 2 and Supplementary Table S2). The 157 non-pancreatic GI-NENs presented with grading information in 54 cases, while the NEC or NET status was known for 46 GI-NENs, of which 31 were NECs or annotated as ambiguous. The six panNEN or GEP-NEN datasets with available grading and NEC or NET annotation exhibited a strong intra and inter-dataset imbalance: Diedisheim [17], Missiaglia [29], Sadanandam [15], and Scarpa [16] were skewed towards low- to medium-grade NETs, while Fröhling [28] and Riemer (Riemer, P.; Otto, R.; Detjen, K.M., et al. Correspondence to pamela.riemer@charite.de, Laboratory of Molecular Tumor Pathology and Systems Biology, Institute of Pathology, Charité Universitätsmedizin Berlin, 10117 Berlin, Germany. Manuscript in preparation) were skewed towards NECs. Moreover, a technological bias was present: Missiaglia [29] utilized a custom array, Sadanandam [15] a generic mRNA array, and the remaining five datasets the bulk RNA-seq technology with the additional limitation that the Diedisheim [17] dataset was limited to the expression data of 9000 genes.

### 3.3. Deconvolution of panNEN and Non-Pancreatic GEP-NEN Transcriptomes into Endocrine and Exocrine-Like Cell Type Proportions

**Figure 3:**
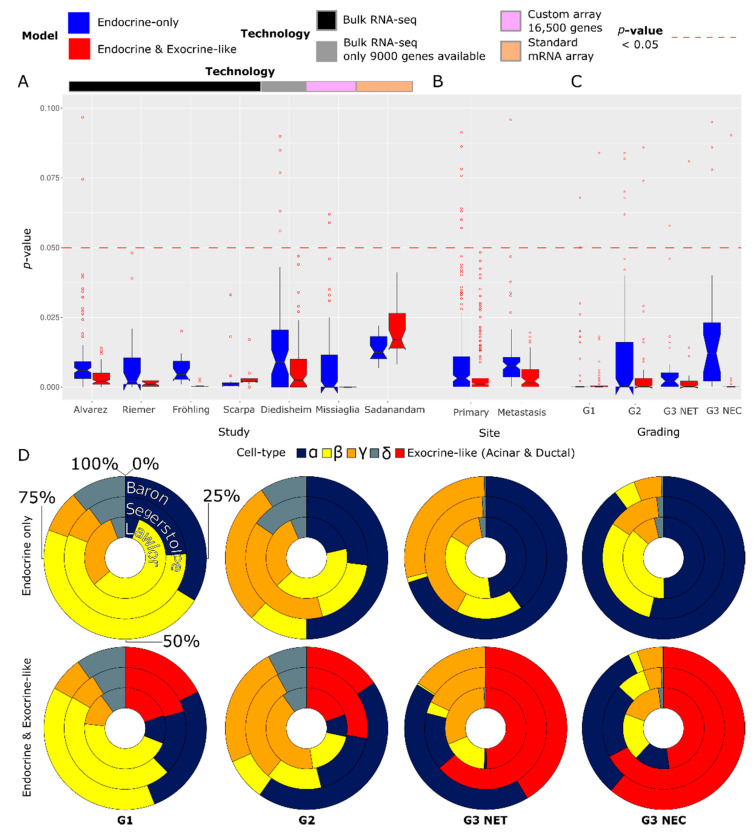
Deconvolution *p*-values and predicted relative cell type proportions. Plots (**A**–**C**) show the deconvolution *p*-value distributions aggregated by study of origin, primary or metastasis status, grading and NEC or NET status. Plot (**D**) shows the relative cell type proportion prediction for the endocrine-only (upper row) and the endocrine exocrine-like mixed model (lower row) aggregated over different gradings and the NEC or NET status.

The deconvolution results were analyzed separately for the panNENs and non-pancreatic NENs of the gastrointestinal tract (GI-NENs) (see Figure 3 for panNENs and Supplementary Figure S1 for non-pancreatic GI-NENs). The statistical power of a deconvolution, as measured by the empirical *p*-value, differed greatly between different gradings, deconvolution models, combinations of the respective algorithm, and the scRNA training dataset as well as the underlying sequencing technology.

We could significantly deconvolve non-neoplastic control samples (overall *p*-value = ~<1 × 10^−8^) and G1 (mean *p*-value = ~1 × 10^−5^) and G2 (mean *p*-value = ~1 × 10^−3^) panNENs, regardless of their status as primary or metastasis, with the endocrine-only model (Supplementary Table S3). However, the endocrine-only model could not deconvolve most G3 NECs and partially high-grade G3 NETs. The mixed endocrine and exocrine-like cell type model could, in contrast, significantly and robustly deconvolve G3 NETs and NECs with three out of the four scRNA cell type training datasets. The mixed model also achieved the significant deconvolution of low-grade panNENs, independently from their grading, site, or study of origin, but was less successful in the deconvolution of the non-neoplastic Fadista [27] dataset. Unlike the superior performance of the mixed model in G3 panNEN, the improvement compared to the endocrine model was marginal for G1- and G2-graded panNENs (see Supplementary Table S3). Deconvolution models trained on the Tosti scRNA training dataset could effectively deconvolve low- to medium-grade paNENs but, unlike the other three scRNA datasets, were unable to effectively deconvolve the majority of NECs and, to a lesser extent, G3 NETs even with the mixed model.

Subsequently, we ranked every combination of the scRNA dataset and algorithm based on their suitability to deconvolve panNENs and GEP-NENs regardless of their grading and identified the combination of BSeq-sc and the Baron et al. [22]. The scRNA dataset is most suited based on the statistical power of the Pearson product–moment correlation *p*-values (see Supplementary Table S4A). If not stated otherwise, all the following results were obtained with this combination.

In addition to the grading, we observed that the *p*-values differed based on the technology applied to generate the underlying sequencing data. Overall, the bulk RNA-sequencing technology was more readily deconvolved than the data generated with mRNA arrays. Moreover, the number of genes present in the data was found to have an impact as well: the deconvolution of the 9000 gene Diedisheim bulk RNA-seq dataset showed a lower statistical power than the four full-transcriptome bulk RNA-seq studies.

### 3.4. Cell Type Proportion Predictions Differ by Grading, Study, and Deconvolution Model

Next, we analyzed the cell type proportion predictions of all seven panNEN datasets based on the scRNA cell type training datasets, which could significantly deconvolve G3 NETs and NECs, i.e., Baron [22], Segerstolpe [24], and Lawlor [23]. We found the predictions of the endocrine-only model for G1 NETs to be similar to the healthy cell types since they approximately resembled the expected cell type stratification observed in healthy pancreatic endocrine tissue [22]. The cell type proportions were found to vary depending on the grading of the neoplasms: the endocrine-only model altered its predictions from a comparatively balanced cell type proportion prediction for low-grade panNENs to a prediction dominated by a single-cell type for the high-grade G3 panNENs, which in case of the endocrine-only model was the α cell type (see Figure 3).

Analogously to the endocrine-only model, the mixed endocrine and exocrine-like model predicted the cell type composition of G1 panNENs as highly similar to the healthy islet stratification, with the exocrine-like cell type proportions only ranging from 15% to 21%. The α cell type proportion was found to increase from low- to high-grade panNEN for the mixed model. However, the exocrine-like cell type proportion increased at an even greater rate than the α cell type proportion regardless of the scRNA training and panNEN benchmark dataset.

### 3.5. Biological Contextualization of the Deconvolution Model Effectiveness and Cell Type Proportions

In view of the high exocrine cell type fractions predicted for G3 NETs, the ineffective deconvolution when using training data from the Tosti [25] study with its deliberate emphasis on the best possible representation of exocrine cell populations seemed counterintuitive. We therefore inspected the protocols used by the scRNA studies to obtain single-cell or -nuclei preparations for sequencing. The protocols stated that the Baron [22], Segerstolpe [24], and Lawlor et al. [23] studies used islets and allowed cells to recover from their isolation before being processed for sequencing, thereby exposing the cells to a period of deliberate in vitro culturing. Different from the islet cells, acinar cells exposed to in vitro culturing are reported to undergo trans-differentiation, referred to as acinar to ductal metaplasia (ADM) [60,61,62,63], conceivably impinging on the marker gene sets of acinar and ductal cell types. In contrast, Tosti et al. [25] processed their cells immediately with minimal potential for ADM, consequently resulting in a lower percentage of acinar and ductal trans-differentiation-associated marker genes. To quantify the representation of trans-differentiation features in the exocrine cell type signatures, we calculated the overlap between the cell type marker genes and the sets of genes annotated by Schlesinger et al. [64] as being involved in the trans-differentiation processes of murine pancreatic acinar cells towards alternative cell types (see Supplementary Table S4B) in an oncogene-driven mouse model of ADM. We found that the extent of the overlap was positively correlated with the deconvolution performance as measured by the *p*-value for the subgroup of NECs and high-grade NETs. The most effective Baron model possessed the highest percentage of trans-differentiation-associated marker genes, Segerstolpe [24] was ranked second with respect to its suitability and it also had the second highest percentage, analogously followed by Lawlor [23], and, lastly, by Tosti [25] with the lowest overlap and suitability to deconvolve high-grade panNENs (see Supplementary Table S4A,B).

Further GSEA analyses probed whether Baron-deconvolved panNENs with an above-average exocrine-like cell type proportion prediction showed a statistically significant similarity to the Schlesinger et al. [64] trans-differentiation clusters relative to below-average exocrine-like panNENs. We discovered a significant enrichment (*p*-value: <10^−6^) of the exocrine-like high panNENs for a murine gene set of acinar-derived cells undergoing trans-differentiation into an intermediate ductal-like cell type (‘cluster A0 ductal-like’ of the Schlesinger et al. [64] study) (see Supplementary Figure S2A). Next, we explored the biological context of the trans-differentiation gene expression profiles in high-grade panNENs and noted reports regarding a small subpopulation of nonmalignant acinar cells called ‘acinar edge’ cells. This acinar cell subpopulation features ADM and progenitor-associated expression profiles with the activation of multiple oncogenic pathways in the absence of oncogenic mutations or evidence of injury [59]. We procured the set of 100 genes with a greater differential upregulation in the ‘acinar edge’ relative to the remaining acinar cells and found a significant enrichment of these genes in panNENs whose exocrine-like cell type proportion predictions were above the average relative to the below average panNEN subgroup (*p*-value: 0.008) (see Supplementary Figure S2B).

Since the lineage plasticity of the trans-differentiating cells was furthermore reported as being connected to the reactivation of stem cell features, we generated a deconvolution model trained on human intestinal stem cells (HISC) in lieu of the exocrine-like cell type with scRNA data from Haber et al. [26] to observe whether stemness-related signature genes similarly improved the deconvolution of NEC and high-grade NET transcriptomes. The HISC model could deconvolve panNENs and GI-NEN and showed a comparable suitability to deconvolve NECs but was subsequently found to be less suited than the exocrine-like mixed model with respect to the derivation of the clinical characteristics of panNEN, which was why we excluded it from further analyses.

The prediction of increasing α cell type proportions with higher grading fits with the recent suggestions to stratify sporadic panNET based on their similarity to α or β-cells, respectively, [14,65,66] with the expression of the α-cell-specific transcription factor ARX in the more advanced stage panNET (see Supplementary Text S1 for an extended biological contextualization).

### 3.6. Correlation of Predicted Cell Type Proportions with Prognostic and Clinical Characteristics

Next, we analyzed whether the deconvolution-derived results were correlated with the clinical prognosis, cell proliferation rate, and overall patient survival time. To that end, we determined how well the mixed model or the HISC model cell type marker genes could cluster our NEN samples. A principal component analysis (PCA) revealed that the marker genes separated NETs and NECs into different clusters, i.e., the marker genes were statistically suited to differentiate NETs from NECs and ambiguous NEN (see Supplementary Figure S3). Next, we analyzed the relationship between the deconvolution predictions and the Sadanandam et al. gene set signature [15]. This signature specifies distinct molecular subgroups for panNENs and identifies panNENs with an increased metastatic potential. We observed common clustering patterns between the cell type proportion predictions, NEC or NET status, and the clustering pattern of panNENs based on those genes that are part of the Sadanandam et al. [15] classification scheme signature (see Supplementary Figure S4). However, the exocrine-like cell type proportion predictions allowed the assignment of subclusters to NENs based on the Sadanandam et al. [15] classification scheme which was not possible based on the proliferation rates alone since these were indistinguishable between the subclusters.

**Figure 4:**
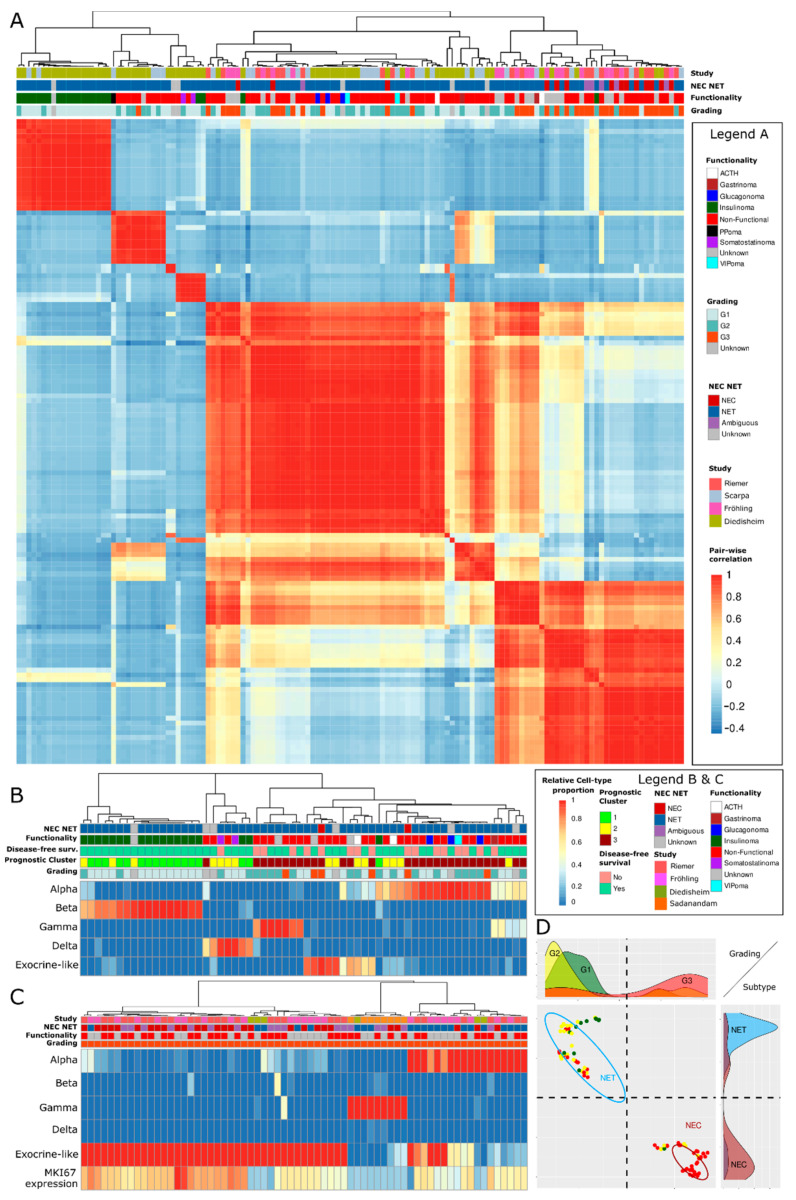
Overview of the relationship between deconvolution predictions and clinical characteristics. Plot (**A**): heatmap of the pairwise correlations of deconvolution results of four Bulk-RNA-seq sequencing datasets. It is shown that the panNENs do not cluster according to their study of origin but according to their NEC or NET and functionality characteristics and, to a lesser extent, grading. Plot (**B**): the deconvolution results of the low- to medium-grade Diedisheim dataset. It is shown that functional characteristics such as the classification as insulinoma are congruent with the deconvolution predictions and that the prognostic clusters (3 worst, 1 best) annotated by Diedisheim et al. [17] correlate with a grouping based on deconvolution results. Disease-free survival state relates to a five-year time point. Plot (**C**): clustering heatmap of 67 high-grade G3 NETs and NECs with both pancreatic and non-pancreatic origin from all four datasets with NECs based on their cell type prediction. It is shown that NECs and NETs are predominantly assigned to different clusters (top left versus right cluster) regardless of their site origin or dataset, which makes their separation and classification possible, while *MKI67* expression correlates with the cell type proportion predictions. Plot (**D**): UMAP projection of GEP-NENs from the Riemer, Scarpa, and Fröhling cohorts onto a two-dimensional surface according to their respective distance in the higher-dimensional space of deconvolution cell type proportions, reconstruction error, *p*-value, and correlation. A linear separability of NENs with different grading and NEC or NET status is possible within the space of deconvolution features.

We verified that the cell type proportion predictions did not cluster panNENs according to their study of origin but according to their clinically relevant properties (Figure 4A). Interestingly, panNENs with an identical grading were partially deconvolved differently and therefore assigned to different deconvolution clusters, indicating a further subtyping that extends beyond the grading. Upon the analysis of the Diedisheim dataset, we ascertained visually that the prognostic clusters assigned to panNEN by Diedisheim et al. [17] matched well with the clusters generated by the deconvolution of the panNEN (Figure 4B). Likewise, the deconvolution-derived endocrine cell type assigned to functional and mostly low-grade Diedisheim panNENs predominantly agreed with their classification, for instance, for insulinoma. We analyzed whether G3 NETs could be discerned from NECs exclusively by clustering them according to their relative cell type proportions and whether a separation was both possible for pancreatic as well as GI-NENs. To that end, we clustered the 67 panNENs and GI-NENs from datasets which included NECs and found that a separation of NEC and NETs via cluster-assignment was possible in the majority of cases regardless of an NENs study of origin or site of the primary tumor (Figure 4C). To increase the statistical power, we next analyzed whether NETs and NECs could be more effectively separated by non-linear uniform manifold approximation and projection (UMAP) [67], which projected 128 panNENs and GI-NENs with NEC or NET annotation from the same four datasets into a lower-dimensional space, which allowed for their linear separation (Figure 4D).

Analyses with a focus on the *MKI67* expression levels and exocrine-like cell type proportions revealed a significant Pearson product–moment correlation between exocrine-like cell type proportions and *MKI67* expression levels for three out of six panNEN datasets (range significant *p*-values: 1 × 10^−3^ to 4 × 10^−3^) (see Supplementary Table S4A for these and the following statistics). Upon a closer inspection, we discovered that the insignificantly correlated dataset had almost indiscernible *MKI67* expression levels (Missiaglia [29]) or were strongly skewed towards either G3 NECs (Fröhling [28]) or low-grade functional panNENs (Diedisheim [17]).

Thereafter, we determined the correlation between the model’s exocrine-like cell type proportion predictions and the histopathology-derived grading information for the six panNEN datasets, which provided the required annotation. The cell type proportion predictions were significantly correlated with the proliferation-associated grading in all six datasets (*p*-value minimum 1 × 10^−4^, maximum 4 × 10^−2^), although only 5% of the exocrine-like marker genes utilized for the proportion predictions were associated with the proliferation activity. Subsequent analysis of variance (ANOVA) tests uncovered that the exocrine-like cell type proportions could effectively separate G3 from G2 and G1 panNENs (*p*-value minimum 4 × 10^−6^, maximum 3.5 × 10^−2^) while G1 panNENs could generally not be effectively discerned from G2 panNENs. As a comparative baseline, we calculated the *MKI67* proliferation marker correlations with the grading and observed significant *p*-values with a slightly stronger but overall similar statistical power (*p*-value minimum 4 × 10^−7^, maximum 1 × 10^−3^) compared to the exocrine-like cell type proportions.

We further quantified the extent to which disaggregated exocrine-like cell types were correlated with grading and found that the statistical power of the ductal cell type was significantly greater than that of the acinar cell type, although their aggregation as an exocrine-like cell type remained superior with respect to the predictive power. As mentioned above, the cells assigned to the ductal lineage in our training datasets were characterized by a high-level representation of the genes associated with acinar to ductal metaplasia (ADM). This suggested that increased ductal cell type predictions reflected an enhanced lineage plasticity in high-grade NEN.

### 3.7. Machine Learning-Based Prediction of Grading, NEC, or NET Status and Patient Survival Time

Due to the observed potential of discerning panNENs with a different grading and of predicting the NEC or NET status, we scrutinized how well an ML model trained on the deconvolution results could classify panNENs with respect to their clinical–pathological characteristics. We therefore trained a baseline ML model on the expression data of all the genes shared between the six annotated panNEN datasets and on the proliferation rate gold-standard biomarker *MKI67* levels, which determines the grading. We formulated three classification tasks: (1) the separation of low and medium panNENs from high-grade G3 panNENs, (2) the assignment of the precise G1 or G2 or G3 class, and (3) the prediction of the NEC or NET status. We compared the results to a deconvolution model using eight features, namely the α, β, γ, and δ endocrine and the exocrine-like cell type proportions, *p*-value, reconstruction error, and correlation r value. Note that the latter model does not contain proliferation-associated features.

**Figure 5:**
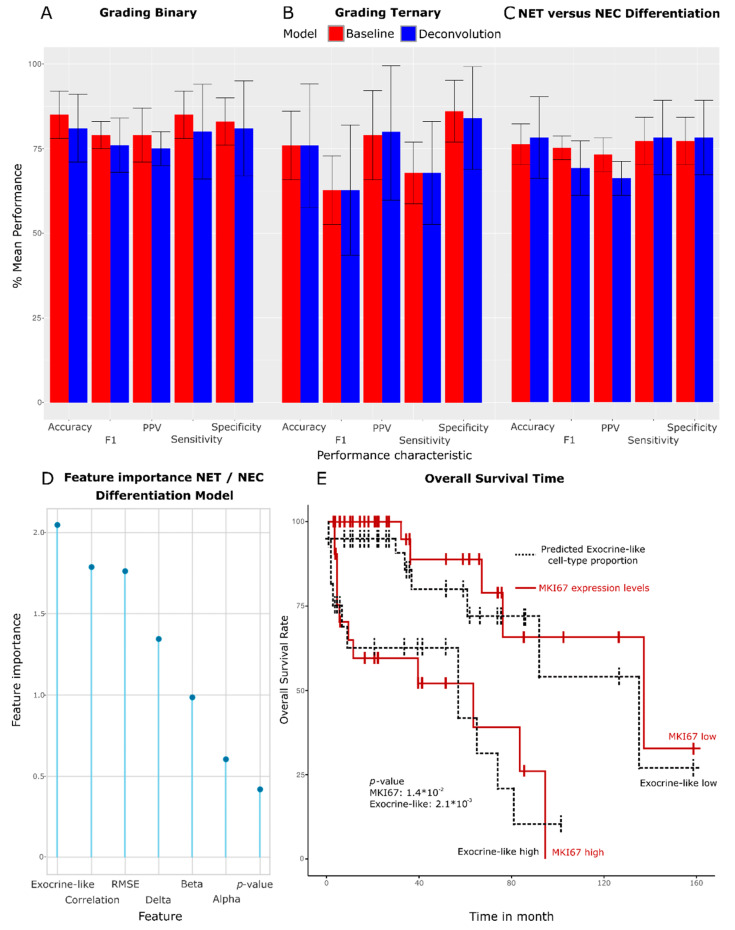
Statistical power of a deconvolution-trained ML model to predict clinically relevant characteristics. Plot (**A**): predictive power of a deconvolution model relative to a baseline model trained on expression data and *MKI67* levels. The baseline model shows slightly higher mean performances, but the performance of either model remains comparable with respect to the discernibility of G3 panNENs from medium- and low-grade G2 and G1 panNENs. Plot (**B**): ternary grading classification performances. The baseline model predicts the specific G1 or G2 or G3 class of a panNEN with a comparable performance as the deconvolution model. Plot (**C**): the performance of the deconvolution model with respect to the classification of a panNEN as either NET or NEC remains comparable to the baseline model with the exception of the F1-score and the positive predictive value (PPV), which is greater in the baseline model. Plot (**D**): feature importance of a deconvolution model tasked with discerning pancreatic NECs from NETs. The exocrine-like cell type proportion was most informative when telling NECs apart from NETs, followed by the quality with which a panNEN could be reconstructed during deconvolution. Endocrine cell type-related properties proved to be of limited relevance. Plot (**E**): Kaplan–Meier plot of the overall survival time of all samples with survival time annotation in the Riemer and Scarpa dataset. *MKI67* baseline as well as the exocrine-like cell type proportion stratified NEN patients into a ‘high’- or ‘low’- risk group membership with comparable Cox hazard ratio test’s *p*-value for this binary task.

In the binary classification task (G1 and G2 versus G3), we observed an accuracy of 85% for the baseline model and 81% for the deconvolution model, a sensitivity of 85% (baseline), 80% (deconvolution), and a positive predictive value (PPV) of 79% (baseline) and 75% (deconvolution) (see Figure 5). The volatility of the deconvolution model’s performance was slightly greater than that of the baseline. The ternary classification task (G1 versus G2 versus G3) revealed balanced accuracies of 75% for both models, class-averaged sensitivities of 85% (baseline) and 83% (deconvolution), and a PPV of 78% (baseline) and 79% (deconvolution).

NEC and NETs could be discerned with accuracies of 76% (baseline) and 78% (deconvolution), sensitivities of 77% (baseline) and 78% (deconvolution), and PPVs of 73% (baseline) and 66% (deconvolution). The ML model interestingly found the exocrine-like cell type proportion to be most useful when discerning NECs from NETs, followed by the reconstruction correlation and error, suggesting that the endocrine cell type properties were of a limited relevance when discerning NECs from NETs (see Figure 5D).

Importantly, the standard deviation of the deconvolution and the baseline model’s accuracy covered the averaged accuracy of the other model’s performance, indicating the comparability of either models’ predictive performance with respect to their accuracy. However, the PPV for the task of discerning NETs from NECs did differ slightly in favor of the baseline model, indicating a limited superiority of the baseline model for this particular task and performance characteristic.

Information on disease-related survival (DRS) was available for two datasets: Riemer and Scarpa. Explorative analyses revealed a statistically significant Pearson Product–moment correlation (r = −0.45, *p*-value 0.017) between the cell type proportions of the 32 high-grade GEP-NENs of the Riemer dataset and their corresponding DRS. We analyzed the survival time prediction performances for the separated Riemer and Scarpa datasets, respectively, as well as their combination into a single dataset to increase the sample size (see Figure 5 and Supplementary Table S4A). We utilized two different cohort designs for the survival tests. The first design used three subgroups (‘low’-, ‘medium’-, and ‘high’-risk subgroups) while the second cohort design was tested on two subgroups (a combined ‘low’- and ‘medium’- subgroup versus a ‘high’- risk subgroup). The three-arm design was chosen to reflect the established ternary clinical standards, while the two-arm design was tested because the previous ANOVA tests indicated that G3 panNENs could be well discerned from G2 panNENs, but not G2 panNENs from G1 panNENs.

The Cox hazard ratio tests revealed that the exocrine-like cell type proportion achieved significance for all three datasets for the binary design (*p*-value minimum 0.006, maximum 0.041) and the ternary design (*p*-value minimum 0.008, maximum 0.02). The statistical power of a *MKI67* baseline model was comparable to that of the exocrine-like cell type proportion in the three-arm (*p*-value minimum 0.0026, maximum 0.049) and the two-arm design (*p*-value minimum 0.0036, maximum 0.014). The grading ground truth showed the greatest statistical power for every design (*p*-value minimum 0.0005, maximum 0.036). We therefore deemed the predictive power of deconvolution-trained models comparable to that of a model trained on the *MKI67* expression levels on the ground of their comparable test statistics while simultaneously finding the statistical power of a ground-truth model trained on the pathologist-derived grading superior to both in silico models with respect to the prediction of a patient’s overall survival time.

## 4. Discussion

Various publications have shown that transcriptomic deconvolution can deconvolve transcriptomes from healthy tissue accurately [41,43,44] and, to a lesser extent, also those of the data derived from neoplastic tissue [68,69,70,71,72]. The first reason to apply a deconvolution approach for the classification of panNENs was that aforementioned publications indicated a relationship between the clinically relevant phenotypes and the deconvolution results. Second, the training of a deconvolution model does not require training data derived from neoplastic tissue but instead only requires widely available data derived from healthy tissue, which counteracted the notorious scarcity of the suited training data for pancreatic and non-pancreatic NENs. The approach was then structured as a two-step ML framework whose first deconvolution step delivered the input for the second step, which predicted the clinical characteristics.

A significant deconvolution of G1 and G2 pancreatic and non-pancreatic NETs was possible regardless of the scRNA training dataset, deconvolution algorithm, or site or study of origin, respectively, suggesting a high relative resemblance of low- to medium-grade panNETs to fully differentiated endocrine cell types (see Figure 3). NECs and partially G3 NETs could not be significantly deconvolved by means of exclusively endocrine cell types, indicating a low resemblance to the fully differentiated endocrine cell types that the deconvolution was based on. However, a mixed model that included exocrine-like cell types in addition to endocrine cell types could effectively deconvolve NEC and partially G3 NETs. The performance of the mixed model was related to the representation of trans-differentiation-associated genes in the exocrine marker gene sets, suggesting an enhanced lineage plasticity of high-grade NEN. Furthermore, an ML model tasked to discern NECs from NETs found the exocrine cell type properties and reconstruction quality to be significantly more relevant for the task than the properties associated with endocrine cell types. These findings suggest that NECs and partially high-grade G3 NETs differ with respect to the exocrine-like, trans-differentiation-associated properties from low- to medium-grade NETs.

A direct quantification of the soundness of panNEN deconvolution remains challenging since no gold-standard dataset exists that would qualify a deconvolution result as correct apart from cases such as, e.g., insulinomas, where deconvolution predominantly provided correct classifications, and an empirical deconvolution *p*-value. Moreover, deconvolution models tend to be susceptible for volatility of results depending on the choice of deconvolution algorithm and the training dataset [41] (see Supplementary Table S4 Sheet A). Therefore, biological interpretability is critical and was achieved by conciliating the exocrine-like aspects with the current understanding of panNEN biology in the related Results section (see Supplementary Text S1 for an extended contextualization).

We assessed whether the deconvolution method could replicate the existing proliferation rate-oriented classification schematics while simultaneously introducing a novel non-proliferation rate-based classification approach for panNENs. To the same end, we compared the predictive performance of a proliferation-independent deconvolution ML model with the established proliferation rate-oriented model (trained on the transcriptome of panNENs as well as the proliferation rate biomarker *MKI67*). We assessed how well the grading, NEC, or NET subtype status and patient overall survival time could be predicted. The proliferation rate model’s predictive performance was found to be comparable for all the aforementioned clinical characteristics, with the slightly greater performance of the proliferation rate model relative to the deconvolution model when differentiating between three types of grading (G1 versus G2 versus G3) (see Figure 5). The slight superiority was, however, anticipated because ANOVA tests revealed beforehand that deconvolution results can efficiently discern between G2 versus G3 NENs but not as well between G1 and G2 NENs. Exocrine-like cell type proportion levels could furthermore add information to the panNETassigner-based classification proposed by Sadanandam et al. [15], since subclusters enriched for NEC emerged (see Supplementary Figure S4).

The deconvolution approach clustered and thereby classified panNENs not by their proliferation rate and grading, respectively, but according to independent molecular mechanisms. PanNENs generally clustered according to their functionality and NEC or NET status but not exclusively by their grading, with G3 panNENs being split between clusters (see Figure 4). Furthermore, a deconvolution model could discern NEC and NET without being informed of the proliferation rate, which suggests that deconvolution can refine the classification scheme of panNENs from the perspective of the panNENs functionality, degree of dedifferentiation, and the origin of the cell type (see Figure 4D). We therefore see the application of the deconvolution approach in particular for the purposes of differentiating between medium- versus high-grade NENs and G3 NETs versus NECs in cases of non-informative proliferation rate measurements, which is also the use-case, where we see the greatest need for an ML-based support of pathologists in case of ambiguity. Further research is required to fully exploit the potential of the deconvolution approach with a deepened understanding of how the clinical characteristics between the deconvolution-derived panNEN classes and clusters differ.

## 5. Conclusions

The combination of transcriptomic deconvolution and ML modeling for the study of panNENs and non-pancreatic GEP-NENs yielded clinically meaningful classifications. Our proposed strategy reduces the dependency on scarcely available neoplastic training data for panNENs and non-pancreatic GEP-NEN research in general. We also believe that this strategy could as well be applied to other rare cancers, as long as the base cell types are known and the scRNA data of these are available. Classification-by-deconvolution has the potential to support pathologists with informative and complementary ML model predictions in cases of an incongruous or uncertain grading and differentiation, which in turn may lead to a better personalization of the clinical management of pancreatic and non-pancreatic NEN patients.

## Figures and Tables

**Table 1:** Name, purpose with respect to transcriptomic deconvolution, source, and reference number of the respective dataset.

Name	Type	Purpose	ID—Source	Reference
Baron	scRNAseq	Training	GSE84133, GEO	[22]
Califano	bulk RNAseq	Benchmark	GSE98894, GEO	[18]
Diedisheim	bulk RNAseq	Benchmark	DOI: 10.1530/ERC-21-0051	[17]
Fadista	bulk RNAseq	Out-group test	GSE50244, GEO	[27]
Haber	scRNAseq	HISC Training	GSE92332, GEO	[26]
Lawlor	scRNAseq	Training	GSE86473, GEO	[23]
Fröhling	bulk RNAseq	Benchmark	EGAS00001004813	[28]
Missiaglia	microarray	Benchmark	GSE73338, GEO	[29]
Riemer	bulk RNAseq	Benchmark	EGAD00001006657	unpublished
Sadanandam	microarray	Benchmark	GSE73339, GEO	[15]
Sato	bulk RNAseq	Benchmark	JGAS000237, NBDC	[30]
Scarpa	bulk RNAseq	Benchmark	EGAS00001001732, ICGC	[16]
Segerstolpe	scRNAseq	Training	E-MTAB-5061, Array Express	[24]
Tosti	snRNAseq	Training	EGAD00001006396, EGA	[25]

## Data Availability

All data, with the exception of the Riemer et al. dataset, are publicly available. The Riemer dataset can be accessed under the ID EGAD00001006657 on the EGA online repository.

## Supplementary Materials

The following supporting information can be downloaded at: https://www.mdpi.com/article/10.3390/cancers15030936/s1, Figure S1: Deconvolution *p*-values for the 157 non-pancreatic GEP-NEN based on BSeq-sc and Baron scRNA dataset; Figure S2: GSEA enrichment results for Schlesinger et al. transdifferentiation cluster A0 and the Acinar edge differential gene expression set; Figure S3: Principal component analysis of the united NEN transcriptomes of the Riemer and Scarpa datasets reduced to sets of cell type marker genes; Figure S4: Correlation heatmap of the panNENs contained in the Scarpa and Riemer datasets reduced to the Sadanandam et al. classification scheme gene set signature genes; Table S1: Overview of the GEP-NEN and panNEN datasets obtained to train and benchmark the deconvolution framework; Table S2: Stratification and clinical annotations of the NEN and GEP-NEN datasets; Table S3: Results of the deconvolution of the GEP-NEN datasets based on the BSeq-sc framework and Baron et al. scRNA training dataset; Table S4: Gridsearch over different scRNA training datasets with BSeq-sc and the transdifferentiation associated genes per cell type; Text S1: Biological Interpretation; Text S2: Supplementary Methods. References [73,74,75,76,77,78,79,80,81,82] are cited in the supplementary materials.

## References

[1] Di Sanzo M., Cipolloni L., Borro M., La Russa R., Santurro A., Scopetti M., Simmaco M., Frati P. (2017). Clinical Applications of Personalized Medicine: A New Paradigm and Challenge. Curr. Pharm. Biotechnol..

[2] Iqbal M.J., Javed Z., Sadia H., Qureshi I.A., Irshad A., Ahmed R., Malik K., Raza S., Abbas A., Pezzani R. (2021). Clinical Applications of Artificial Intelligence and Machine Learning in Cancer Diagnosis: Looking into the Future. Cancer Cell Int..

[3] Rindi G., Wiedenmann B. (2020). Neuroendocrine Neoplasia of the Gastrointestinal Tract Revisited: Towards Precision Medicine. Nat. Rev. Endocrinol..

[4] Sorbye H., Welin S., Langer S.W., Vestermark L.W., Holt N., Osterlund P., Dueland S., Hofsli E., Guren M.G., Ohrling K. (2013). Predictive and Prognostic Factors for Treatment and Survival in 305 Patients with Advanced Gastrointestinal Neuroendocrine Carcinoma (WHO G3): The NORDIC NEC Study. Ann. Oncol..

[5] Dasari A., Mehta K., Byers L.A., Sorbye H., Yao J.C. (2018). Comparative Study of Lung and Extrapulmonary Poorly Differentiated Neuroendocrine Carcinomas: A SEER Database Analysis of 162,983 Cases. Cancer.

[6] Dasari A., Shen C., Halperin D., Zhao B., Zhou S., Xu Y., Shih T., Yao J.C. (2017). Trends in the Incidence, Prevalence, and Survival Outcomes in Patients With Neuroendocrine Tumors in the United States. JAMA Oncol..

[7] Basturk O., Yang Z., Tang L.H., Hruban R.H., Adsay V., McCall C.M., Krasinskas A.M., Jang K.-T., Frankel W.L., Balci S. (2015). The High-Grade (WHO G3) Pancreatic Neuroendocrine Tumor Category Is Morphologically and Biologically Heterogenous and Includes Both Well Differentiated and Poorly Differentiated Neoplasms. Am. J. Surg. Pathol..

[8] Tang L.H., Untch B.R., Reidy D.L., O’Reilly E., Dhall D., Jih L., Basturk O., Allen P.J., Klimstra D.S. (2016). Well-Differentiated Neuroendocrine Tumors with a Morphologically Apparent High-Grade Component: A Pathway Distinct from Poorly Differentiated Neuroendocrine Carcinomas. Clin. Cancer Res..

[9] Yachida S., Vakiani E., White C.M., Zhong Y., Saunders T., Morgan R., de Wilde R.F., Maitra A., Hicks J., Demarzo A.M. (2012). Small Cell and Large Cell Neuroendocrine Carcinomas of the Pancreas Are Genetically Similar and Distinct from Well-Differentiated Pancreatic Neuroendocrine Tumors. Am. J. Surg. Pathol..

[10] Elvebakken H., Perren A., Scoazec J.-Y., Tang L.H., Federspiel B., Klimstra D.S., Vestermark L.W., Ali A.S., Zlobec I., Myklebust T.Å. (2020). A Consensus Developed Morphological Re-Evaluation of 196 High-Grade Gastroenteropancreatic Neuroendocrine Neoplasms and Its Clinical Correlations. Neuroendocrinology.

[11] Tang L.H., Basturk O., Sue J.J., Klimstra D.S. (2016). A Practical Approach to the Classification of WHO Grade 3 (G3) Well-Differentiated Neuroendocrine Tumor (WD-NET) and Poorly Differentiated Neuroendocrine Carcinoma (PD-NEC) of the Pancreas. Am. J. Surg. Pathol..

[12] Tang L.H. (2020). Pancreatic Neuroendocrine Neoplasms: Landscape and Horizon. Arch. Pathol. Lab. Med..

[13] Simbolo M., Bilotta M., Mafficini A., Luchini C., Furlan D., Inzani F., Petrone G., Bonvissuto D., La Rosa S., Schinzari G. (2021). Gene Expression Profiling of Pancreas Neuroendocrine Tumors with Different Ki67-Based Grades. Cancers.

[14] Cejas P., Drier Y., Dreijerink K.M.A., Brosens L.A.A., Deshpande V., Epstein C.B., Conemans E.B., Morsink F.H.M., Graham M.K., Valk G.D. (2019). Enhancer Signatures Stratify and Predict Outcomes of Non-Functional Pancreatic Neuroendocrine Tumors. Nat. Med..

[15] Sadanandam A., Wullschleger S., Lyssiotis C.A., Grötzinger C., Barbi S., Bersani S., Körner J., Wafy I., Mafficini A., Lawlor R.T. (2015). A Cross-Species Analysis in Pancreatic Neuroendocrine Tumors Reveals Molecular Subtypes with Distinctive Clinical, Metastatic, Developmental, and Metabolic Characteristics. Cancer Discov..

[16] Scarpa A., Chang D.K., Nones K., Corbo V., Patch A.-M., Bailey P., Lawlor R.T., Johns A.L., Miller D.K., Mafficini A. (2017). Whole-Genome Landscape of Pancreatic Neuroendocrine Tumours. Nature.

[17] Diedisheim M., Dermine S., Jouinot A., Septier A., Gaujoux S., Dousset B., Cadiot G., Larger E., Bertherat J., Scharfmann R. (2021). Prognostic Transcriptome Classes of Duodenopancreatic Neuroendocrine Tumors. Endocr. Relat. Cancer.

[18] Alvarez M.J., Subramaniam P.S., Tang L.H., Grunn A., Aburi M., Rieckhof G., Komissarova E.V., Hagan E.A., Bodei L., Clemons P.A. (2018). A Precision Oncology Approach to the Pharmacological Targeting of Mechanistic Dependencies in Neuroendocrine Tumors. Nat. Genet..

[19] Eraslan G., Avsec Ž., Gagneur J., Theis F.J. (2019). Deep Learning: New Computational Modelling Techniques for Genomics. Nat. Rev. Genet..

[20] Schaefer J., Lehne M., Schepers J., Prasser F., Thun S. (2020). The Use of Machine Learning in Rare Diseases: A Scoping Review. Orphanet J. Rare Dis..

[21] Rashid H., Tanveer M.A., Aqeel Khan H. (2019). Skin Lesion Classification Using GAN Based Data Augmentation. Conf. Proc. IEEE Eng. Med. Biol. Soc..

[22] Baron M., Veres A., Wolock S.L., Faust A.L., Gaujoux R., Vetere A., Ryu J.H., Wagner B.K., Shen-Orr S.S., Klein A.M. (2016). A Single-Cell Transcriptomic Map of the Human and Mouse Pancreas Reveals Inter- and Intra-Cell Population Structure. Cell Syst..

[23] Lawlor N., George J., Bolisetty M., Kursawe R., Sun L., Sivakamasundari V., Kycia I., Robson P., Stitzel M.L. (2017). Single-Cell Transcriptomes Identify Human Islet Cell Signatures and Reveal Cell-Type-Specific Expression Changes in Type 2 Diabetes. Genome Res..

[24] Segerstolpe Å., Palasantza A., Eliasson P., Andersson E.-M., Andréasson A.-C., Sun X., Picelli S., Sabirsh A., Clausen M., Bjursell M.K. (2016). Single-Cell Transcriptome Profiling of Human Pancreatic Islets in Health and Type 2 Diabetes. Cell Metab..

[25] Tosti L., Hang Y., Debnath O., Tiesmeyer S., Trefzer T., Steiger K., Ten F.W., Lukassen S., Ballke S., Kühl A.A. (2021). Single-Nucleus and In Situ RNA–Sequencing Reveal Cell Topographies in the Human Pancreas. Gastroenterology.

[26] Haber A.L., Biton M., Rogel N., Herbst R.H., Shekhar K., Smillie C., Burgin G., Delorey T.M., Howitt M.R., Katz Y. (2017). A Single-Cell Survey of the Small Intestinal Epithelium. Nature.

[27] Fadista J., Vikman P., Laakso E.O., Mollet I.G., Esguerra J.L., Taneera J., Storm P., Osmark P., Ladenvall C., Prasad R.B. (2014). Global Genomic and Transcriptomic Analysis of Human Pancreatic Islets Reveals Novel Genes Influencing Glucose Metabolism. Proc. Natl. Acad. Sci. USA.

[28] Horak P., Heining C., Kreutzfeldt S., Hutter B., Mock A., Hüllein J., Fröhlich M., Uhrig S., Jahn A., Rump A. (2021). Comprehensive Genomic and Transcriptomic Analysis for Guiding Therapeutic Decisions in Patients with Rare Cancers. Cancer Discov..

[29] Missiaglia E., Dalai I., Barbi S., Beghelli S., Falconi M., della Peruta M., Piemonti L., Capurso G., Di Florio A., delle Fave G. (2010). Pancreatic Endocrine Tumors: Expression Profiling Evidences a Role for AKT-mTOR Pathway. J. Clin. Oncol..

[30] Kawasaki K., Toshimitsu K., Matano M., Fujita M., Fujii M., Togasaki K., Ebisudani T., Shimokawa M., Takano A., Takahashi S. (2020). An Organoid Biobank of Neuroendocrine Neoplasms Enables Genotype-Phenotype Mapping. Cell.

[31] Schneider V.A., Graves-Lindsay T., Howe K., Bouk N., Chen H.-C., Kitts P.A., Murphy T.D., Pruitt K.D., Thibaud-Nissen F., Albracht D. (2017). Evaluation of GRCh38 and de Novo Haploid Genome Assemblies Demonstrates the Enduring Quality of the Reference Assembly. Genome Res..

[32] Patro R., Duggal G., Love M.I., Irizarry R.A., Kingsford C. (2017). Salmon Provides Fast and Bias-Aware Quantification of Transcript Expression. Nat. Methods.

[33] Love M.I., Anders S., Kim V., Huber W. (2015). RNA-Seq Workflow: Gene-Level Exploratory Analysis and Differential Expression. F1000 Res..

[34] Bray N.L., Pimentel H., Melsted P., Pachter L. (2016). Near-Optimal Probabilistic RNA-Seq Quantification. Nat. Biotechnol..

[35] Haider S., Ballester B., Smedley D., Zhang J., Rice P., Kasprzyk A. (2009). BioMart Central Portal—Unified Access to Biological Data. Nucleic Acids Res..

[36] Durinck S., Spellman P.T., Birney E., Huber W. (2009). Mapping Identifiers for the Integration of Genomic Datasets with the R/Bioconductor Package biomaRt. Nat. Protoc..

[37] Sing T., Sander O., Beerenwinkel N., Lengauer T. (2005). ROCR: Visualizing Classifier Performance in R. Bioinformatics.

[38] Subramanian A., Tamayo P., Mootha V.K., Mukherjee S., Ebert B.L., Gillette M.A., Paulovich A., Pomeroy S.L., Golub T.R., Lander E.S. (2005). Gene Set Enrichment Analysis: A Knowledge-Based Approach for Interpreting Genome-Wide Expression Profiles. Proc. Natl. Acad. Sci. USA.

[39] Shen-Orr S.S., Tibshirani R., Khatri P., Bodian D.L., Staedtler F., Perry N.M., Hastie T., Sarwal M.M., Davis M.M., Butte A.J. (2010). Cell Type-Specific Gene Expression Differences in Complex Tissues. Nat. Methods.

[40] Moffitt R.A., Marayati R., Flate E.L., Volmar K.E., Loeza S.G.H., Hoadley K.A., Rashid N.U., Williams L.A., Eaton S.C., Chung A.H. (2015). Virtual Microdissection Identifies Distinct Tumor- and Stroma-Specific Subtypes of Pancreatic Ductal Adenocarcinoma. Nat. Genet..

[41] Dong M., Thennavan A., Urrutia E., Li Y., Perou C.M., Zou F., Jiang Y. (2021). SCDC: Bulk Gene Expression Deconvolution by Multiple Single-Cell RNA Sequencing References. Brief. Bioinform..

[42] Wang N., Gong T., Clarke R., Chen L., Shih I.-M., Zhang Z., Levine D.A., Xuan J., Wang Y. (2015). UNDO: A Bioconductor R Package for Unsupervised Deconvolution of Mixed Gene Expressions in Tumor Samples. Bioinformatics.

[43] Wang X., Park J., Susztak K., Zhang N.R., Li M. (2019). Bulk Tissue Cell Type Deconvolution with Multi-Subject Single-Cell Expression Reference. Nat. Commun..

[44] Newman A.M., Liu C.L., Green M.R., Gentles A.J., Feng W., Xu Y., Hoang C.D., Diehn M., Alizadeh A.A. (2015). Robust Enumeration of Cell Subsets from Tissue Expression Profiles. Nat. Methods.

[45] Newman A.M., Steen C.B., Liu C.L., Gentles A.J., Chaudhuri A.A., Scherer F., Khodadoust M.S., Esfahani M.S., Luca B.A., Steiner D. (2019). Determining Cell Type Abundance and Expression from Bulk Tissues with Digital Cytometry. Nat. Biotechnol..

[46] Ritchie M.E., Phipson B., Wu D., Hu Y., Law C.W., Shi W., Smyth G.K. (2015). Limma Powers Differential Expression Analyses for RNA-Sequencing and Microarray Studies. Nucleic Acids Res..

[47] Yoshihara K., Shahmoradgoli M., Martínez E., Vegesna R., Kim H., Torres-Garcia W., Treviño V., Shen H., Laird P.W., Levine D.A. (2013). Inferring Tumour Purity and Stromal and Immune Cell Admixture from Expression Data. Nat. Commun..

[48] Kuhn M., Johnson K. (2019). Feature Engineering and Selection: A Practical Approach for Predictive Models.

[49] Tritschler S., Theis F.J., Lickert H., Böttcher A. (2017). Systematic Single-Cell Analysis Provides New Insights into Heterogeneity and Plasticity of the Pancreas. Mol. Metab..

[50] Bonner-Weir S., Inada A., Yatoh S., Li W.-C., Aye T., Toschi E., Sharma A. (2008). Transdifferentiation of Pancreatic Ductal Cells to Endocrine Beta-Cells. Biochem. Soc. Trans..

[51] Puri S., Folias A.E., Hebrok M. (2015). Plasticity and Dedifferentiation within the Pancreas: Development, Homeostasis, and Disease. Cell Stem. Cell.

[52] Yachida S., Totoki Y., Noe M., Nakatani Y., Horie M., Kawasaki K., Nakamura H., Saito-Adachi M., Suzuki M., Takai E. (2022). Comprehensive Genomic Profiling of Neuroendocrine Carcinomas of the Gastrointestinal System. Cancer Discov..

[53] Konukiewitz B., Jesinghaus M., Steiger K., Schlitter A.M., Kasajima A., Sipos B., Zamboni G., Weichert W., Pfarr N., Klöppel G. (2018). Pancreatic Neuroendocrine Carcinomas Reveal a Closer Relationship to Ductal Adenocarcinomas than to Neuroendocrine Tumors G3. Hum. Pathol..

[54] Venizelos A., Elvebakken H., Perren A., Nikolaienko O., Deng W., Lothe I.M.B., Couvelard A., Hjortland G.O., Sundlöv A., Svensson J. (2021). The Molecular Characteristics of High-Grade Gastroenteropancreatic Neuroendocrine Neoplasms. Endocr. Relat. Cancer.

[55] Simon T., Riemer P., Detjen K., Di Domenico A., Bormann F., Menne A., Khouja S., Monjé N., Childs L.H., Lenze D. (2021). DNA Methylation Reveals Distinct Cells of Origin for Pancreatic Neuroendocrine Carcinomas (PanNECs) and Pancreatic Neuroendocrine Tumors (PanNETs). bioRxiv.

[56] Qadir M.M.F., Álvarez-Cubela S., Klein D., Lanzoni G., García-Santana C., Montalvo A., Pláceres-Uray F., Mazza E.M.C., Ricordi C., Inverardi L.A. (2018). P2RY1/ALK3-Expressing Cells within the Adult Human Exocrine Pancreas Are BMP-7 Expandable and Exhibit Progenitor-like Characteristics. Cell Rep..

[57] Qadir M.M.F., Álvarez-Cubela S., Klein D., van Dijk J., Muñiz-Anquela R., Moreno-Hernández Y.B., Lanzoni G., Sadiq S., Navarro-Rubio B., García M.T. (2020). Single-Cell Resolution Analysis of the Human Pancreatic Ductal Progenitor Cell Niche. Proc. Natl. Acad. Sci. USA.

[58] Grün D., Muraro M.J., Boisset J.-C., Wiebrands K., Lyubimova A., Dharmadhikari G., van den Born M., van Es J., Jansen E., Clevers H. (2016). De Novo Prediction of Stem Cell Identity Using Single-Cell Transcriptome Data. Cell Stem Cell.

[59] Gopalan V., Singh A., Rashidi Mehrabadi F., Wang L., Ruppin E., Arda H.E., Hannenhalli S. (2021). A Transcriptionally Distinct Subpopulation of Healthy Acinar Cells Exhibit Features of Pancreatic Progenitors and PDAC. Cancer Res..

[60] Baldan J., Houbracken I., Rooman I., Bouwens L. (2019). Adult Human Pancreatic Acinar Cells Dedifferentiate into an Embryonic Progenitor-like State in 3D Suspension Culture. Sci. Rep..

[61] De Lisle R.C., Logsdon C.D. (1990). Pancreatic Acinar Cells in Culture: Expression of Acinar and Ductal Antigens in a Growth-Related Manner. Eur. J. Cell Biol..

[62] Storz P. (2017). Acinar Cell Plasticity and Development of Pancreatic Ductal Adenocarcinoma. Nat. Rev. Gastroenterol. Hepatol..

[63] Giroux V., Rustgi A.K. (2017). Metaplasia: Tissue Injury Adaptation and a Precursor to the Dysplasia-Cancer Sequence. Nat. Rev. Cancer.

[64] Schlesinger Y., Yosefov-Levi O., Kolodkin-Gal D., Granit R.Z., Peters L., Kalifa R., Xia L., Nasereddin A., Shiff I., Amran O. (2020). Single-Cell Transcriptomes of Pancreatic Preinvasive Lesions and Cancer Reveal Acinar Metaplastic Cells’ Heterogeneity. Nat. Commun..

[65] Chan C.S., Laddha S.V., Lewis P., Koletsky M., Robzyk K., Da Silva E., Torres P.J., Untch B., Bose P., Chan T.A. (2018). ATRX, DAXX or MEN1 Mutant Pancreatic Neuroendocrine Tumors Are a Distinct Alpha-Cell Signature Subgroup. Nat. Commun..

[66] Di Domenico A., Pipinikas C.P., Maire R.S., Bräutigam K., Simillion C., Dettmer M.S., Vassella E., Thirlwell C., Perren A., Marinoni I. (2020). Epigenetic Landscape of Pancreatic Neuroendocrine Tumours Reveals Distinct Cells of Origin and Means of Tumour Progression. Commun Biol..

[67] McInnes L., Healy J., Saul N., Großberger L. (2018). UMAP: Uniform Manifold Approximation and Projection. J. Open Source Softw..

[68] Yoon S.-J., Park J., Shin Y., Choi Y., Park S.W., Kang S.-G., Son H.Y., Huh Y.-M. (2020). Deconvolution of Diffuse Gastric Cancer and the Suppression of CD34 on the BALB/c Nude Mice Model. BMC Cancer.

[69] Thrane K., Eriksson H., Maaskola J., Hansson J., Lundeberg J. (2018). Spatially Resolved Transcriptomics Enables Dissection of Genetic Heterogeneity in Stage III Cutaneous Malignant Melanoma. Cancer Res..

[70] Berglund E., Maaskola J., Schultz N., Friedrich S., Marklund M., Bergenstråhle J., Tarish F., Tanoglidi A., Vickovic S., Larsson L. (2018). Spatial Maps of Prostate Cancer Transcriptomes Reveal an Unexplored Landscape of Heterogeneity. Nat. Commun..

[71] Peng X.L., Moffitt R.A., Torphy R.J., Volmar K.E., Yeh J.J. (2019). De Novo Compartment Deconvolution and Weight Estimation of Tumor Samples Using DECODER. Nat. Commun..

[72] Gentles A.J., Newman A.M., Liu C.L., Bratman S.V., Feng W., Kim D., Nair V.S., Xu Y., Khuong A., Hoang C.D. (2015). The Prognostic Landscape of Genes and Infiltrating Immune Cells across Human Cancers. Nat. Med..

[73] Ma Z., Lytle N.K., Chen B., Jyotsana N., Novak S.W., Cho C.J., Caplan L., Ben-Levy O., Neininger A.C., Burnette D.T. (2022). Single-Cell Transcriptomics Reveals a Conserved Metaplasia Program in Pancreatic Injury. Gastroenterology.

[74] Pan F.C., Bankaitis E.D., Boyer D., Xu X., Van de Casteele M., Magnuson M.A., Heimberg H., Wright C.V.E. (2013). Spatiotemporal Patterns of Multipotentiality in Ptf1a-Expressing Cells during Pancreas Organogenesis and Injury-Induced Facultative Restoration. Development.

[75] Ebrahimi A.G., Hollister-Lock J., Sullivan B.A., Tsuchida R., Bonner-Weir S., Weir G.C. (2020). Beta Cell Identity Changes with Mild Hyperglycemia: Implications for Function, Growth, and Vulnerability. Mol. Metab..

[76] Zhang J., Baran J., Cros A., Guberman J.M., Haider S., Hsu J., Liang Y., Rivkin E., Wang J., Whitty B. (2011). International Cancer Genome Consortium Data Portal--a One-Stop Shop for Cancer Genomics Data. Database.

[77] Athar A., Füllgrabe A., George N., Iqbal H., Huerta L., Ali A., Snow C., Fonseca N.A., Petryszak R., Papatheodorou I. (2019). ArrayExpress Update—From Bulk to Single-Cell Expression Data. Nucleic Acids Res..

[78] Stanescu D.E., Yu R., Won K.-J., Stoffers D.A. (2017). Single Cell Transcriptomic Profiling of Mouse Pancreatic Progenitors. Physiol. Genom..

[79] Yan L., Yang M., Guo H., Yang L., Wu J., Li R., Liu P., Lian Y., Zheng X., Yan J. (2013). Single-Cell RNA-Seq Profiling of Human Preimplantation Embryos and Embryonic Stem Cells. Nat. Struct. Mol. Biol..

[80] Terry M.T., Patricia M. (2001). Grambsch Modeling Survival Data: Extending the Cox Model. Stat. Med..

[81] Gaujoux R., Seoighe C. (2010). A Flexible R Package for Nonnegative Matrix Factorization. BMC Bioinform..

[82] Scholkopf B., Smola A.J., Williamson R.C., Bartlett P.L. (2000). New Support Vector Algorithms. Neural Comput..

