# Supplementary material for: Transcriptomic Deconvolution of Neuroendocrine Neoplasms Predicts Clinically Relevant Characteristics
Source: Cancers (Basel). 2023 Feb 1;15(3):936. doi: 10.3390/cancers15030936 (PMC9913692; doi:10.3390/cancers15030936)
Supplement: Supplementary file 1 [file cancers-15-00936-s001.zip › cancers-2059633 Supplementary Materials.pdf]

cancers

MDPI

Supplementary Materials: Transcriptomic Deconvolution of Neuroendocrine Neoplasms Predicts Clinically Relevant Characteristics

Raik Otto, Katharina M. Detjen, Pamela Riemer, Melanie Fattohi, Carsten Grötzinger, Guido Rindi, Bertram Wiedenmann, Christine Sers and Ulf Leser

Text S1: Biological Interpretation

The model predictions of high exocrine-like cell-type proportions in high-grade panNENs suggested similarities with non-transformed exocrine cells endowed with plasticity and relaxed lineage commitment, as found in the recently described acinar edge population or in ADM. ADM occurs in models of acinar injury and regeneration and furthermore characterizes the preneoplastic pancreatic exocrine lesions in mouse models of PDAC [64]. It may give rise to ductal or endocrine differentiated cells as well as cells resembling extrapancreatic gastric or enteroendocrine cell types [64,73,74]. Conversely, an acquisition of exocrine features by islet cells occurred in a rat model of mild islet cell injury, indicating that ductal characteristics are within the range of lineage plasticity of pancreatic endocrine cells [75]. Hence, the non-endocrine cell type predictions for high-grade GEP-NENs have biological correlates in dedifferentiation or transdifferentiation processes in rodent models. Lineage trees derived in-silico from single-cell sequencing of adult human pancreatic cells moreover assigned ductal cells a central position, with subpopulations giving rise to different endocrine cell-types [58]. Thus, exocrine features of high-grade NEN transcriptomes may alternatively reflect such an endocrine progenitor population, possibly pointing to a 'reserve' multipotency of adult ductal cells. Irrespective of the exact underlying mechanistic scenario, exocrine-like marker genes separated NEN transcriptomes in clusters dominated by NECs and NETs, respectively, as shown in Supplementary Figure S3, further supporting that exocrine-like cell-type predictions relate to biologically relevant features of NENs. In this context, the almost exclusive prediction of exocrine-like cell type proportions in panNEC fits with the proposed acinar origin emerging from methylation data, and with the frequent observation of admixtures of ductal adenocarcinoma portions in pancreatic NECs.

The observed correlation between cell-type proportions and *MKI67* levels is a notable finding, because staining levels of *MKI67* represent the current gold-standard method for panNEN and GEP-NEN grading. A transitive correlation of cell-type proportions was anticipated and found for all six panNEN datasets with grading annotation between the α and in particular exocrine-like cell-type proportions. Furthermore, the relationship between cell-type proportion and grading was remarkable because the deconvolution-trained ML model was not informed about *MKI67* levels while cell-type marker genes were only associated with proliferation activity up to 5%. The limited degree of proliferation-associated marker genes was anticipated, since marker genes are tasked to differentiate between fully differentiated cell-types with comparable proliferation rates i.e. proliferation-associated marker genes would not be informative with respect to cell-type distinction. Therefore, we believe that the deconvolution-trained prediction of characteristics can serve as a complementary approach to the established proliferation-based methods in that information which is not proliferation-rate-derived can be integrated into a ML model to increase its predictive power.

While our study focused on panNENs, the rarity of resection tissues from pancreatic NECs led us to include high-grade non-pancreatic GEP-NENs, which originate from other locations in the gastroenteric system. Therefore, the tissue of origin could affect the deconvolution results. However, the overall performance of the in-silico classification

model remained stable between datasets that contained GEP-NENs of purely pancreatic origin and those that included high-grade samples of diverse gastrointestinal tissue-backgrounds. Furthermore, we ascertained that the endocrine-only and mixed models were not biased for either pancreatic or non-pancreatic tissue by verifying that the marker genes predominantly consisted of genes with similar expression in pancreatic and non-pancreatic NENs. Careful future validation of the deconvolution approach in non pancreatic tissues is nonetheless mandatory, since a recent multi-omics based characterization of a large high-grade NEN cohort [52] reported distinct genomic features of panNEN when compared to tumors from other gastrointestinal sites. An application of the current deconvolution approach in the analysis of the Sato dataset [30] indicates that deconvolution also can be applied to organoid cultures.

Cell-type predictions were correlated with the recurrence-free survival in the dataset provided by Diedisheim et al. [17]. The preferential prediction of β or δ cell features was noted in samples of the prognostically favorable cluster, whereas *α* and exocrine cell-type predictions dominated in samples from the unfavorable cluster, altogether suggesting that deconvolution reflects molecular subtypes that impinge on the clinical course. Indeed, our deconvolution-based models were able to accurately predict the patient overall survival time of GEP-NEN patients in a two-arm and in a three-arm cohort design. Partitioning the united Riemer and Scarpa dataset into an exocrine-like high and an exocrine-like low sub-cohort revealed a significant difference in disease-related survival time, comparable to that of partitioning the cohort by *MKI67* expression and only slightly inferior to a partitioning based on the pathologists-derived grading ground-truth. However, we observed a high degree of variation of the survival-time test's p-value between different deconvolution algorithms and different training sets, indicating a need for further fine-tuning of the method.

Text S2: Supplementary Methods

Source and use of datasets

We procured two panNEN and one mixed, pancreatic non-pancreatic GEP-NEN dataset from the publicly accessible gene omnibus database GEO and obtained the Scarpa et al. dataset from ICGC [76]. The Riemer et al. dataset was made available by C. Grötzinger, Charité Berlin. Seven scRNA deconvolution training datasets were located on publicly available GEO servers with the exception of the Segerstolpe et al. dataset that was acquired from the Array Express database [77]. Three additional scRNA training datasets (Haber [26], Stanescu [78], Yan [79]) were subjected to preliminary benchmarks but not to detailed result analyses based on ranking, which revealed inferior performance for the purpose of GEP-NEN deconvolution. The ranking evaluated whether the deconvolution algorithms were tested on a given datasets, the number of sequenced cells, a stratification of the cell types roughly correlated to the stratification in healthy tissue and the technological homogeneity of the datasets compared to each other in order to facilitate the interpretation of benchmark results.

Determination of algorithm and scRNA training datasets most suited for GEP-NEN deconvolution

We chose BSeq-sc, MuSiC and Moffitt et al. due to their proven ability to deconvolve either healthy pancreatic tissue (BSeq-sc, MuSiC) or cancerous exocrine pancreatic tissue (Moffitt et al. [40]). Subsequently, we identified the combination of training scRNA dataset and deconvolution algorithm whose predictions were most suited by comparing the stability and significance of the resulting correlations. In particular, the correlation with *MKI67* levels averaged over all patient-derived bulk RNA-seq datasets together with the algorithm-specific quality score was taken as a measurement of effectiveness. The Pearson product moment correlations of the relative fractions and the *MKI67* levels were subsequently calculated to compare the performance to predict sample grading and

patient survival, see Supplementary Figure 5. Data was processed according to Love et al. best practice (https://www.bioconductor.org/packages/devel/workflows/vignettes/rnaseqGene/inst/doc/rnaseqGene.html) and GATK best practice guidelines (https://gatk.broadinstitute.org/hc/en-us/sections/360007226651-Best-Practices-Workflows, accessed 10 November 2021).

Software

We ran differential expression analyses via the 'DESeq2' R package where we formulated the design matrix based on cohort and study membership to exclude potential batch effects during differential expression analysis [33,34]. 'Ggplot2' and 'ggbiplot' were utilized for graphics generation. 'Survival', 'sleuth', 'biomaRt' and 'RocR' were further R packages utilized for numeric analyses and the 'stringR' R package for string related operations [37,39,80]. The software 'GSEA' as provided by the Broad Institute, Linux version 4.0.2 was utilized for enrichment analyzes [38]. The survival curves were trained with R-package 'Survminer', version 0.4.8.

The BSeq-sc 1.0 R-implementation algorithm was acquired from https://github.com/shenorrLabTRDF/bseqsc and CIBERSORT from cibersort.stanford.edu (accessed 23 November 2020). Beforehand, the most recent version 1.4 of the csSAM [31] R-package required to run BSeq-sc had to be obtained from github (accessed 11 June 2020). CIBERSORTx was procured from cibersortx.stanford.edu. The MuSiC [43] algorithm version 0.1.1 was obtained from the GitHub repository github.com/xuranw/MuSiC (accessed 10 June 2021). The Moffitt et al. [40] NMF algorithm was trained according to the specifications laid out in the corresponding publication which were replicated with the R-package 'NMF' version 0.22 [81].

Features use of the Deconvolution Algorithms

The extension of the highly cited CIBERSORT algorithm with BSeq-sc framework utilized a ν-support vector regression that was optimized for parsimonious modeling due to the ν parameter which is an upper bound on the training error and lower bound on the relative fraction of support vectors thereby reducing overfitting in contrast to the default C-SVR implementation [82]. MuSiC is based on a NMF and is unique in that no specified marker genes are required due to a dynamic gene weighting that prioritizes informative genes and suppresses information from non-informative genes with a reported ability to discern between closely related tissues such as exocrine and endocrine. The Moffitt et al. [40] NMF algorithm was designed to deconvolve pancreatic ductal carcinomas and thus was benchmarked on panNENs and GEP-NENs. The Moffitt et al. [40] algorithm was implemented as specified in the related publication due to a lack of publicly available implementations.

Generally, G2 NENs appeared to be classifiable with less certainty for either model since two classification thresholds, both G1s and G3s have to be correctly estimated as opposed to G1s and G3s that each only present with one threshold. Analysis of the 'Deconvolution' model revealed that both reconstruction error of a transcriptome, as measured by the RMSE, and the ductal cell-type proportion were the most important features, thereby supporting assumptions *i*) and *ii*) specified in section 'Deconvolution algorithms, base cell-types, and evaluation datasets'. Important features of the 'Expression & MKI67' model were *MKI67* and proliferation or cell-cycle regulating genes.

The NEC versus NET subtype Machine-Learning algorithm applied a logistic regression to distinguish the subtypes based on either the ductal, HISC or *MKI67* expression levels, respectively. In contrast, we applied a 'Random Forest' algorithm to predict the clinical characteristics for both the 'Deconvolution' and 'Expression & MKI67' models and compared the observed predictive performances for each dataset.

The method first projected the input data (expression levels) into the space of deconvolution results (see Figure 3D). The 'training' mentioned in the methods section

refers to this projection which necessitates the training of the 'projecting' (NMF) algorithms on scRNA datasets. The survival time-prediction, in turn ,did not require training itself but the algorithm which produced the input data for the survival-time predicting ML model did require training.

The output of the PyCarret software utilized for the ML column and basis for the prediction of clinical characteristics was: a table with the columns "sample name", "true class", "predicted class" and, if applicable, a column specifying the confidence with which the class was predicted by the algorithm, depending on whether the algorithm has a confidence prediction. Secondly, various performance characteristics are derived from the aforementioned output: A confusion matrix, specifying the aggregated amounts of TPs, TNs, FPs, FNs and various derived statistics such as the shown positive-predictive value, sensitivity (recall), specificity (precision), negative-predicted value, kappa-statistics (how much better than just guessing the largest class are the predictions) and various more.

We determined the optimal amount of genes (50–800) because the amount of genes included in a deconvolution model as marker genes is generally correlated with the performance of a deconvolution model. In the past, collinearity and expression of a gene in more than a single cell-type, respectively, led to statistical problems that older models could not address, thereby requiring to 'pre-select' marker genes that were e.g. only expressed in a single cell-type. 'Modern' algorithms such as CIBERSORT are capable of addressing collinearity and ambiguous expression patterns, therefore abolishing the need to pre-select marker genes. The naive approach would therefore be to 'feed' as many genes as possible to a deconvolution algorithm, assuming that the algorithm would select the right marker genes, which may in a frequent amount of cases be true. However, RAM and calculation-time constraints remain. Therefore, a compromise between optimal marker-gene selection and these constraints has to be found.

We addressed the overfitting of the ML model via cross-validation during the training time with an 80% training to 20% testing dataset while measuring the performance on the validation/hold out dataset as implemented in the PyCarret software (version 3.0).

Conception of the baseline versus deconvolution model approach

The baseline versus deconvolution model approach followed the assumption that more features allow to explain more variance if the features are independently and identically distributed (i.i.d.)—but not necessarily gaussian—i.e., it was assumed that a larger model—as measured by the amount of features—should outperform the smaller model unless the smaller model is qualitatively better because it can explain a comparable amount of variance with significantly fewer features with higher information content. The naive assumption that the larger model can perform better is shown in the results where the larger baseline model either performs equally well or slightly better. However, as can be seen, the smaller (deconvolution model) performs similarly well than the significantly larger baseline model which allows the interpretation that the smaller model condenses the statistical information into much more informative features i.e., is by Occam's razor superior. The key consideration was, that the smaller (deconvolution) model was not only smaller, but it was also utilizing independent, orthogonal information to predict clinical characteristics compared to the baseline model, thereby, leading to new insights and generating a complementary approach to the established approach (e.g., Ki-67 staining as proliferation rate marker).

Out-group tests, and superiority over simpler methods

We performed three sanity tests to verify the correlation of *MKI67* levels with predicted cell-type fractions.

Firstly, we tested whether the effect is cancer-specific i.e., absent in healthy tissue. Indeed, deconvolution of the healthy control dataset (Fadista et al.) [27] lacked a correlation of *MKI67* levels, ductal and HISC fractions. Secondly, we determined that a

differential expression analysis in conjunction with a generalized linear model predicted the *MKI67* levels with comparable (2×) or worse (3×) statistical power. Given that a logistic model was superior in only one dataset (Missiaglia), the differential expression-based model was discarded because of inferior predictive power. Thirdly, we determined whether the correlation could be caused by confounding contamination with stromal cells, specifically immune-cells. However, ESTIMATE analysis [47] of the RepSet revealed no correlation of stromal or immune scores with annotated clinical parameters or deconvolution outcomes.

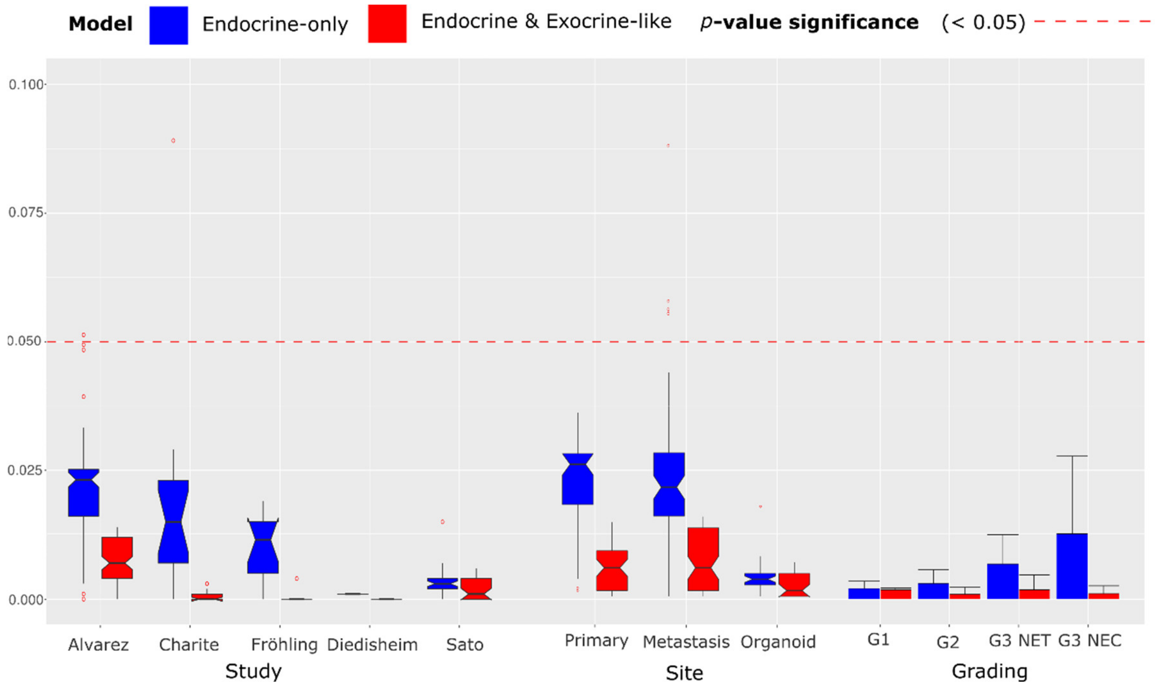

Figure S1. Deconvolution p-values for the 157 non-pancreatic GEP-NEN based on BSeq-sc and the Baron scRNA dataset. The BSeq-sc algorithm trained on Baron scRNA cell-type training data could significantly deconvolve GEP-NEN regardless of their study-of-origin (**A**) site of their primary (**B**) or grading (**C**). The mixed endocrine exocrine-like model always deconvolved with greater statistical power than the endocrine-only model.

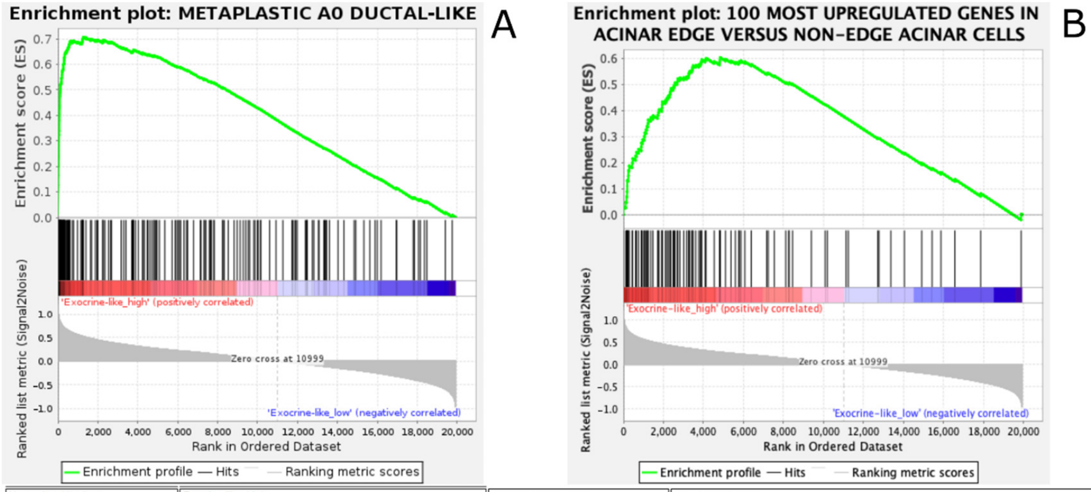

Upregulated in class	Exocrine-like_high
GeneSet	METAPLASTIC A0 DUCTAL-LIKE
Enrichment Score (ES)	0.70686054
Normalized Enrichment Score (NES)	2.209891
Nominal p-value	0.0
FDR q-value	0.0017374734
FWER p-Value	0.002

Upregulated in class	Exocrine-like_high
GeneSet	100 MOST UPREGULATED GENES IN ACINAR EDGE VERSUS NON-EDGE ACINAR CELLS
Enrichment Score (ES)	0.6035765
Normalized Enrichment Score (NES)	1.8708788
Nominal p-value	0.002074689
FDR q-value	0.04123066
FWER p-Value	0.126

Figure S2. GSEA enrichment results for Schlesinger et al transdifferentiation cluster A0 and the Acinar edge differential gene expression set. The significant enrichment of the transdifferentiation-associated ductal-like cell-type gene set 'A0 ductal-like' in the exocrine-like high relative to the exocrine-like low panNENs is shown in subplot (**A**). Subplot (**B**) shows the enrichment of the same exocrine-like high subgroup for the set of the 100 genes whose expression was differentially upregulated the greatest in the edge acinar state compared to the non-edge acinar state according to Gopalan et al. [59].

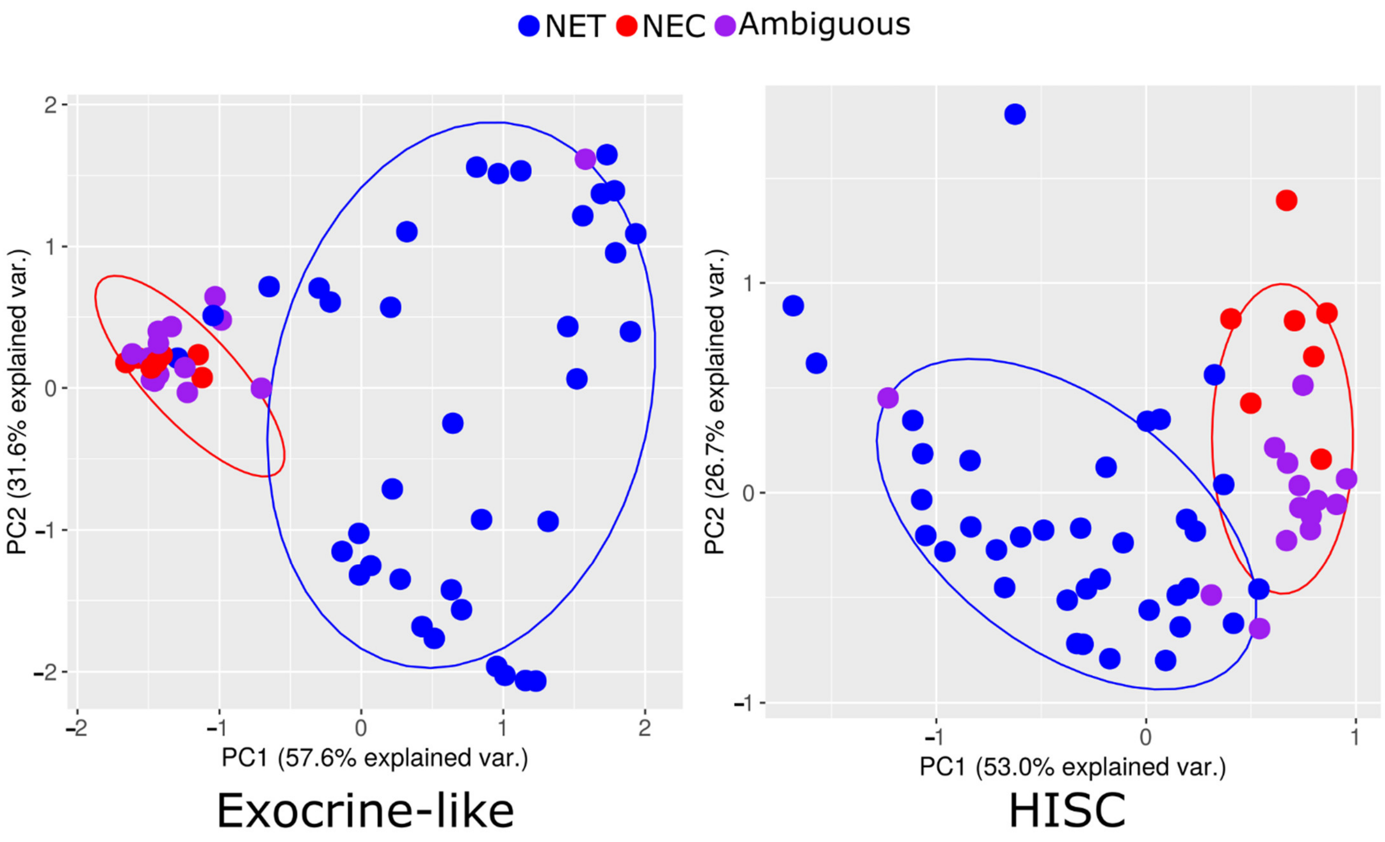

Figure S3. Principal component analysis of the united NEN transcriptomes of the Riemer and Scarpa datasets reduced to sets of cell type marker genes. Expression datasets were reduced to the marker gene sets of the exocrine-like and HISC cell types as determined by the BSeq-sc algorithm, respectively, and their PCAs depicted after colorization for NEC and NET subtype. Purple dots represent samples whose NEC or NET classification was not unanimously possible. It is visualized that a ML model trained on the exocrine-like or HISC signature-based deconvolution results can classify NECs and NETs because NECs and NETs differ characteristically in the sets of genes that are utilized to deconvolve the exocrine-like or HISC cell type proportions. No clustering by tissue of origin was identified, which was supported by the finding that the underlying exocrine-like and HISC marker genes predominantly consist of genes whose expression does not differ between the relevant tissue type of origin.

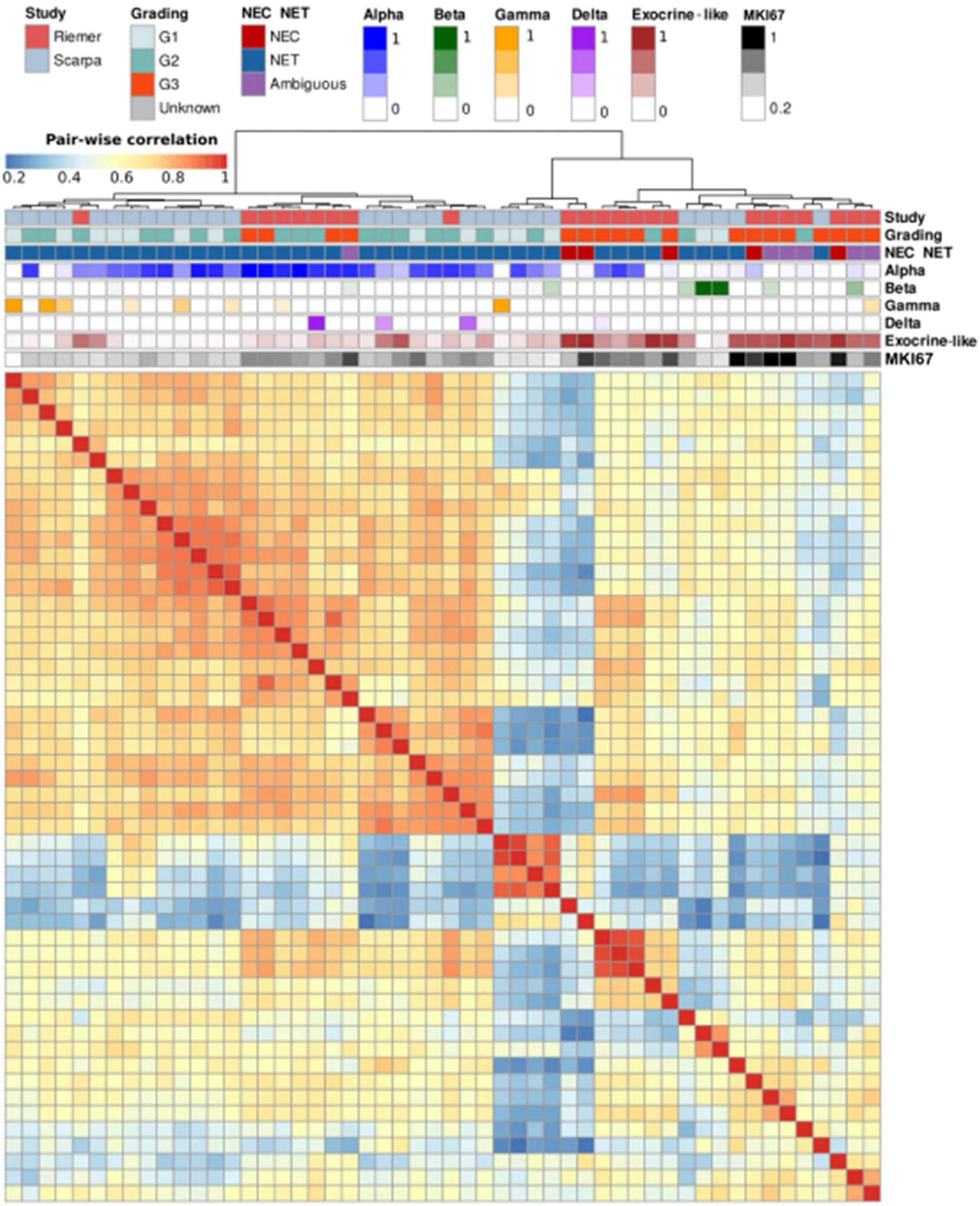

Figure S4. Correlation heatmap of the panNENs contained in the Scarpa and Riemer datasets reduced to the Sadanandam et al. [15] classification scheme gene set signature genes. The correlation heatmap shows that a reduction to the Sadanandam et al. signature genes induces a clustering of the NENs whose cell-type proportion predictions follow the same clustering pattern as the one introduced by the Sadanandam et al. [15] signature. It is therefore shown that the cell-type proportion predictions allow for a classification of a NEN as a member of a Sadanandam et al. [15] class based on the deconvolution of the NEN. Furthermore, it is shown that the α cell-type proportion of the mixed model is anticorrelated with the exocrine-like cell-type proportion and that *MKI67* expression levels are positively correlated with the exocrine-like cell-type proportion predictions.

Table S1 (separate Excel file): Overview of the GEP-NEN and panNEN datasets obtained to train and benchmark the deconvolution framework.

Table S2 (separate Excel file): Stratification and clinical annotations of the NEN and GEP-NEN datasets.

Table S3 (separate Excel file): Results of the deconvolution of the GEP-NEN datasets based on the BSeq-sc framework and Baron et al. scRNA training dataset.

Table S4 (separate Excel file): Gridsearch over different scRNA training datasets with BSeq-sc and the transdifferentiation associated genes per cell type.
